# Graph and Geometric Deep Learning for Intracranial Aneurysm Geometry and Hemodynamics: A Systematic Review with Implications for Neurovascular Implant Design and Evaluation

**DOI:** 10.3390/life16091538

**Published:** 2026-09-15

**Authors:** Rudolfh Batista Arend, Bruno Zilli Peroni, Natan Lucca Lima, Miguel Cruz Garcia, Rafael Torres Fonseca dos Santos, Daniel Kerpel, Gustavo Simiano Jung, Alex Roman, Guilherme Gago, Martin Batista Coutinho da Silva, Antonio Delacy Martini Vial, Edoardo Agosti

**Affiliations:** 1 Faculty of Medicine, Federal University of Fronteira Sul, Passo Fundo 99010-200, RS, Brazil; rudolfharend77@gmail.com (R.B.A.);; 2 Faculty of Medicine, Federal University of Santa Catarina, Araranguá 88905-120, SC, Brazil; 3 Faculty of Medicine, Institute of Medical Education, Rio de Janeiro 20071-004, RJ, Brazil; 4 Faculty of Medicine, Federal University of Santa Maria, Santa Maria 97105-900, RS, Brazil; 5 Department of Neurosurgery, Neurological Institute of Curitiba, Curitiba 82821-020, PR, Brazil; 6 Department of Neurosurgery, Université Laval, Québec, QC G1V 0A6, Canada; 7 Department of Medicine, University of Passo Fundo, Passo Fundo 99052-900, RS, Brazil; 8 Department of Neurosurgery, Irmandade Santa Casa de Misericórdia de Porto Alegre, Hospital São José, Porto Alegre 90020-090, RS, Brazil; 9 Division of Neurosurgery, Department of Medical and Surgical Specialties, Radiological Sciences and Public Health, University of Brescia, 25121 Brescia, Italy

**Keywords:** biomaterials, tissue engineering, neurosurgery, intracranial aneurysm, flow diverter, endothelialization, graph neural network, geometric deep learning, computational fluid dynamics, wall shear stress

## Abstract

**Background:** Neurosurgery depends heavily on implanted biomaterials, and the endovascular treatment of intracranial aneurysms (IAs) is the paradigmatic case, since coils, flow diverters and intrasaccular devices achieve durable occlusion only through intrasaccular thrombus organization and endothelial coverage of the neck, both governed by the local flow environment. Computational fluid dynamics (CFD) resolves that environment but is too slow and too solver-dependent for clinical or device design use. Graph and geometric deep learning operates natively on the unstructured meshes in which vascular anatomy and implanted scaffolds are represented and has been proposed as the technology that would close this gap. **Methods:** Four databases were searched up to 29 July 2026 for original studies developing or validating graph-based or geometric deep learning applied to IA geometry, with a hemodynamic or clinical outcome. Screening and extraction were performed in duplicate; risk of bias with PROBAST and artificial intelligence signaling items, reporting with TRIPOD+AI, and synthesis followed SWiM. **Results:** Twelve studies (2021 to 2026) were included: seven clinical (1965 aneurysms, plus 81 externally) and five computational (up to 984 geometries). Learned geometric representations discriminated rupture, growth and post-embolization recanalization better than morphological indices and then PHASES, reaching an area under the curve of 0.795 to 0.97 internally. Graph and point cloud surrogates reproduced hemodynamic fields with normalized errors of 2% to 5% in seconds rather than hours, and one transformer-based graph network predicted the extent and timing of intra-aneurysmal thrombus formation over a reactive surface. External validation was reached by two studies, and clinical utility was achieved in one; discrimination fell from 0.85 to 0.71 in one external test, and surrogate error rose from 4.1% to 19.1% on patient-derived anatomy. No model was trained on a device-laden geometry. Additionally, 5/7 clinical studies classified cross-sectional rupture status rather than prospectively predicting future rupture. **Conclusions:** Graph and geometric deep learning has substantially reduced the computational burden of hemodynamic analysis, but reliable generalization to unseen patient-specific anatomy remains a relevant challenge. Shared patient-specific benchmarks, prespecified external validation, reporting of calibration, and extension of training corpora to implanted anatomy are necessary next steps towards virtual evaluation of neurovascular biomaterials.

## 1. Introduction

Few fields of neurosurgery are as dependent on implanted materials as the endovascular treatment of intracranial aneurysms (IAs). These focal dilations of the cerebral arteries, most of them arising at or near the circle of Willis, are present in roughly 2% to 5% of the adult population [1,2]. The overwhelming majority never bleed, but when rupture occurs, the consequences are disproportionate to the prevalence of the lesion, since aneurysmal subarachnoid hemorrhage carries a case fatality of approximately one third and leaves a substantial proportion of survivors with permanent cognitive or motor deficit [3,4]. Because incidental cross-sectional imaging now detects aneurysms with increasing frequency, the clinical problem has shifted from finding them to deciding which of them to treat, and with what.

The second half of that question is a biomaterials question. Preventive clipping and endovascular treatment carry nontrivial procedural morbidity [5,6,7], and the devices themselves have proliferated: detachable platinum coils, hydrogel and bioactive coatings, braided nitinol and cobalt chromium flow diverters, and intrasaccular flow disruptors. None of these repair the diseased arterial wall. Each installs a scaffold that reshapes the flow entering the sac, and each depends for durable exclusion of the aneurysm on the same two tissue-level events, organization of intrasaccular thrombus into fibrous tissue and migration of endothelial cells across the neck to construct a neointima on the implanted material [8,9]. Occlusion, in other words, is a tissue engineering outcome obtained with a foreign scaffold, and its characteristic failure modes, incomplete occlusion and post-treatment recanalization, are failures of that construct rather than of the metal. Recanalization after coil embolization was documented in a fifth to a third of treated aneurysms in the early angiographic follow-up series [10], and although packing density explains part of that variation [11], it explains only part, which is precisely why the flow environment inside the treated sac has remained an object of study for two decades.

What determines whether the construct succeeds, and what determines whether an untreated aneurysm remains stable, is largely the same variable. Wall shear stress (WSS) is the dominant mechanical signal read by the vascular endothelium, and it sets endothelial phenotype, junctional integrity and the inflammatory and matrix remodeling program downstream of it [12]. What ruptures is a wall that has lost its endothelial lining, its smooth muscle cellularity and much of its organized collagen [13], and in resected human aneurysm domes the intraluminal flow conditions reconstructed from preoperative angiography correspond to the histological grade of degeneration and inflammatory infiltration [14]. Hemodynamic descriptors derived from computational fluid dynamics (CFD), namely WSS, its time average, the oscillatory shear index and the relative residence time of blood within the sac, have accordingly been associated with rupture status, growth and wall degeneration across multiple cohorts [15,16,17,18,19]. The risk instruments that actually guide practice, the PHASES score for rupture, the ELAPSS score for growth and the multidisciplinary UIATS consensus, contain none of this information, being built almost entirely on aneurysm size, location and a handful of patient covariates, with discrimination typically in the region of an area under the curve (AUC) of 0.70 [20,21,22].

The reason the mechanistic descriptors have not displaced the demographic ones is not that they are wrong; it is that they cannot be obtained in time. A single transient, patient-specific simulation requires hours of computation, meshing expertise and a dedicated software environment; the successive international CFD challenges showed that independent groups solving the same geometry arrive at appreciably different WSS fields depending on solver settings, rheological assumptions, wall behavior and boundary conditions [23,24,25]. In vivo measurement of aneurysmal flow, for instance by four-dimensional flow magnetic resonance imaging, remains too coarse to arbitrate between competing simulations [26]. Patient-specific hemodynamic modeling is therefore a textbook example of an experimental innovation that has not translated simultaneously the most mechanistically plausible and the least clinically used of the available descriptors, and the same barrier confines virtual evaluation of neurovascular implants to a handful of scenarios chosen in advance.

Machine learning offers a way past that barrier. If a model is trained once, offline, on a corpus of high-fidelity simulations, inference on a new geometry becomes almost instantaneous, and the heavy computation is paid only once. Early attempts used convolutional neural networks, which assume a regular grid; cerebral vasculature is not a regular grid, and interpolating an unstructured tetrahedral mesh onto a voxel lattice discards precisely the near-wall detail and local connectivity that determine WSS. Graph neural networks and, more broadly, geometric deep learning were built for this setting. Message passing architectures propagate information along the edges of the computational mesh itself, respecting adjacency and tolerating arbitrary resolution [27,28,29]; point cloud networks of the PointNet family achieve permutation-invariant encoding of unordered surface samples [30,31]; mesh-based simulators learn the temporal evolution of a physical field directly on the simulation graph [32,33]; and the wider geometric deep learning program supplies the symmetry and equivariance arguments that justify these choices [34]. Physics-informed and physics-constrained variants add residuals of the governing equations to the loss, reducing the volume of training data required [35]. Adjacent vascular beds have shown what this buys: SE(3)-equivariant mesh networks approximate directional WSS on coronary surfaces with errors of a few per cent and speedups of up to two orders of magnitude [36], and a gauge-equivariant mesh convolutional network trained on more than a thousand real coronary arteries preserved the association between WSS and myocardial infarction that CFD-derived WSS provides [37]. Public resources specific to the intracranial circulation, notably the IntrA aneurysm dataset and the AnXplore fluid–structure interaction corpus, have made comparable work possible in the cerebral vasculature [38,39].

Existing systematic reviews have addressed either conventional CFD methodology or feature-based machine learning built on clinical and morphological covariates [18,19,40,41,42,43]. None has appraised graph and geometric deep learning as a methodological class, none has asked how faithfully these models reproduce the CFD reference they are intended to replace, and none has examined what the technology implies for the design and evaluation of the implanted materials on which neurovascular practice now rests. We, therefore, conducted a systematic review with four questions in view. First, how accurately do these models reproduce high-fidelity hemodynamic fields, and at what computational gain? Second, how well do they discriminate clinically meaningful endpoints, and how does that discrimination behave under external validation? Third, how far along the path from experimental innovation to clinical translation has each study traveled? And fourth, what would be required to extend these models from the untreated aneurysm to the device-laden one, where they would inform the design and virtual testing of neurovascular biomaterials?

## 2. Materials and Methods

### 2.1. Protocol, Registration and Reporting

The review was conducted according to a protocol prepared in advance and registered with the International Prospective Register of Systematic Reviews (PROSPERO, CRD420261464236). Reporting follows the Preferred Reporting Items for Systematic Reviews and Meta-Analyses (PRISMA) 2020 statement [44], and the search is reported according to the PRISMA-S extension [45]. Departures from the protocol, if any, are declared in the corresponding section below.

### 2.2. Eligibility Criteria

Eligibility was framed as a PICO(TS) question, summarized in Supplementary Table S1.

Population. Human intracranial or cerebral aneurysms, ruptured or unruptured, represented as three-dimensional vascular models derived from computed tomography angiography (CTA), magnetic resonance angiography (MRA), three-dimensional rotational angiography (3DRA) or digital subtraction angiography (DSA). In silico cohorts assembled from patient-derived geometries were eligible. Idealized or parametrically generated geometries were eligible when the study also reported patient-derived cases or when the study explicitly quantified transfer to patient-derived anatomy, and such studies were recorded separately and analyzed as a prespecified subgroup. Extracranial aneurysms and non-aneurysmal vascular disease were excluded.

Intervention. Any graph-based or geometric deep learning model applied to the aneurysm geometry or to its computational mesh, including message passing graph neural networks, graph convolutional networks, graph attention networks, graph transformers, MeshGraphNet-type simulators, spectral graph convolutional networks, gauge or SE(3)-equivariant mesh networks, point cloud architectures of the PointNet family, and physics-informed or physics-constrained variants of any of these. The model was required to output a hemodynamic field or a parameter derived from one, a rupture-related risk estimate, or another aneurysm-specific clinical endpoint. Convolutional neural networks, U-Net architectures, multilayer perceptrons, and classical machine learning models without a graph or point set component were not eligible as index models but were eligible as comparators.

Comparator. At least one of the following: a high-fidelity finite volume or finite element CFD or multiphysics simulation of the same geometry, serving as reference standard for hemodynamic accuracy; an established clinical or morphological risk instrument; a non-graph machine learning baseline; or observed clinical status. Studies reporting no quantitative comparison against any reference were excluded.

Outcomes. The primary outcomes were, first, agreement with the reference simulation for hemodynamic fields and derived parameters, expressed as coefficient of determination, mean absolute error, normalized mean absolute error, root mean square error, relative L2 error, correlation or spatial overlap; and second, discrimination for a clinical endpoint, expressed as AUC, accuracy, sensitivity, specificity or calibration. Secondary outcomes were inference time relative to the reference solver, generalization to unseen geometries and to unseen inflow conditions, training set size, the presence and result of external validation, and the public availability of data and code.

Timing and study design. Any original prediction model development or validation study, including retrospective patient cohorts, in silico benchmark studies and methodological papers reporting quantitative performance. Narrative and systematic reviews, editorials, commentaries and conference abstracts without extractable performance data were excluded. No date or language restriction was applied, although the field is bounded in practice by the introduction of modern message passing architectures.

### 2.3. Information Sources and Search Strategy

PubMed and MEDLINE, Embase, Scopus and the Web of Science Core Collection were searched from inception to 29 July 2026. The strategy combined three concept blocks: intracranial aneurysm; graph-based and geometric deep learning; and hemodynamics, CFD or rupture risk, with Boolean AND. The third block was deliberately broad and merged mechanistic with clinical vocabulary, because a substantial part of this literature originates in engineering groups whose papers never use the word rupture. Controlled vocabulary was used where available, recognizing that the relevant computational terminology is only partially represented in MeSH and Emtree. The full strategies for all four databases are reproduced verbatim in Supplementary Table S2 and were peer-reviewed against the PRESS checklist before execution [46]. Supplementary searching comprised backward and forward citation tracking of all included studies, hand searching of preprint servers and of the proceedings of the principal medical imaging and machine learning conferences, and inspection of the repositories associated with the public aneurysm datasets.

### 2.4. Selection Process

Records were deduplicated and uploaded to Rayyan (Rayyan Systems Inc., Cambridge, MA, USA) [47]. Two reviewers independently screened titles and abstracts, and then independently assessed the full text of all records retained at that stage, with disagreement resolved by discussion and, where necessary, by a third reviewer. Independent duplicate screening was used to reduce selection error and improve the reliability of study selection [48]. Reasons for exclusion were recorded for every full text assessed.

### 2.5. Data Extraction

A structured extraction form was piloted on two included studies and refined before full extraction. Two reviewers extracted data independently, and discrepancies were reconciled against the source article. Extracted items covered study identification and design, country, imaging modality, cohort size in patients and in aneurysms, comparison groups, age and sex distribution, aneurysm location and size, input representation and feature set, model architecture, comparators, validation scheme, primary endpoint, reported performance, external validation, reference standard, key findings, and author-stated limitations. For studies using a CFD reference, we additionally extracted solver, rheological model, wall assumption, boundary conditions, mesh density and the number of simulated cardiac cycles, since these determine the reference against which accuracy is measured. Data and code availability were extracted as an explicit item. Where a report contained internal numerical inconsistencies, the inconsistency was recorded rather than silently resolved.

### 2.6. Risk of Bias and Reporting Quality

Risk of bias and applicability were assessed with the Prediction Model Risk of Bias Assessment Tool (PROBAST) across its four domains: participants, predictors, outcome and analysis [49], supplemented by the signaling items developed for artificial intelligence-based prediction models [50]. Heterogeneity of the reference standard was treated as an additional, prespecified domain, on the grounds that each surrogate study generates its own CFD ground truth under solver settings that are known to influence the result. Reporting completeness was appraised against TRIPOD+AI [51], and deployability against the FUTURE-AI consensus principles [52]. Two reviewers appraised each study independently.

### 2.7. Translational Stage Classification

To address the question of how far each line of work has traveled from experimental innovation towards clinical use, we classified every included study post hoc on a four-stage descriptive ladder, using only information already recorded in the extraction form. Stage 1 denotes development and evaluation on internal data alone, whether by a held-out split or by cross-validation, without independent validation and without explicit robustness testing. Stage 2 adds documented robustness testing, meaning evaluation under boundary conditions, inflow waveforms or geometries deliberately excluded from training. Stage 3 denotes independent external validation, meaning quantitative evaluation in a cohort or dataset not used at any point in development. Stage 4 denotes a demonstrated effect on clinician decision-making or on a patient-relevant outcome. This classification is descriptive rather than evaluative; it was not part of the registered protocol, and it does not replace the formal risk of bias assessment.

### 2.8. Synthesis

Meta-analysis was not appropriate and was not attempted. Accuracy was reported on non-commensurable scales, against reference simulations that differ between studies, and for endpoints that range from a continuous physical field to a binary clinical label; pooling such estimates would produce a summary without interpretable meaning. Synthesis was therefore narrative and tabular, following the reporting guideline for synthesis without meta-analysis [53]. Studies were grouped by the function the model performs, namely geometric encoding for clinical classification as against surrogate modeling of the CFD solution, and, within the second group, by whether the geometries were patient-derived or parametrically generated. Results are presented as reported by the original authors, with the units and metrics they used, and no derived or recalculated statistics are presented except where explicitly identified as such.

## 3. Results

### 3.1. Study Selection

**Figure 1:**
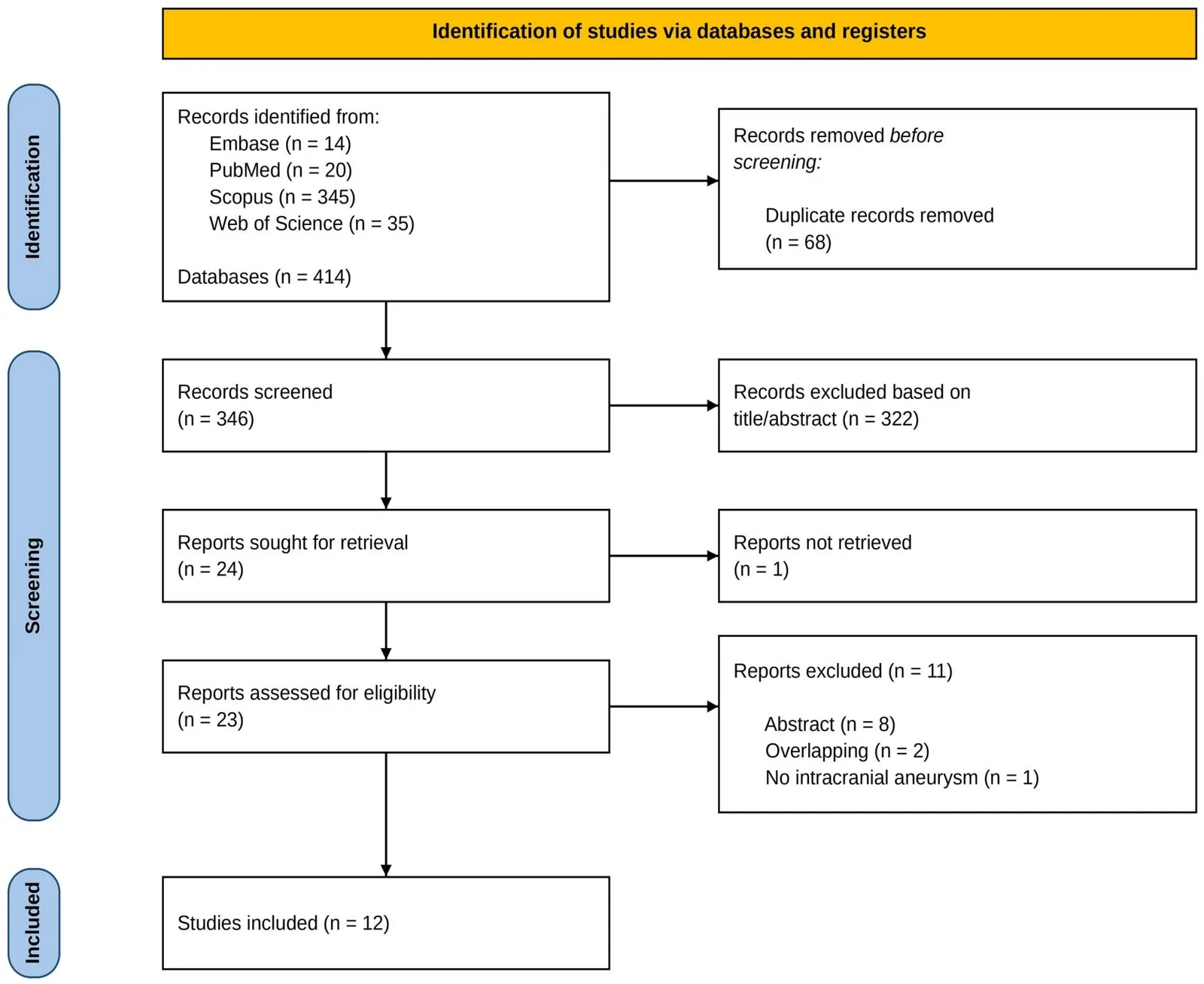
PRISMA 2020 flow diagram of study identification, screening and inclusion.

The database searches returned 414 records, 20 from PubMed, 14 from Embase, 345 from Scopus and 35 from Web of Science. After removal of 68 duplicates, 346 records were screened at title and abstract, of which 322 were excluded. Twenty-four reports were sought for retrieval, and one could not be obtained, leaving 23 assessed in full text. Eleven were excluded, eight because they were conference abstracts without extractable performance data, two because they reported overlapping cohorts already represented by a more complete report, and one because the population did not include intracranial aneurysms. Twelve studies were included in the synthesis [54,55,56,57,58,59,60,61,62,63,64,65]. The selection process is shown in Figure 1.

### 3.2. Characteristics of Included Studies

The twelve included studies were published between 2021 and 2026, and ten of them appeared from 2023 onwards, so that the greater part of this literature is less than three years old. Geographically, the evidence is concentrated: five studies originated in China [55,56,63,64,65], three in France and all three from the same research center [58,59,61], and one each in Slovenia [54], South Korea in collaboration with Slovenia [57], Japan [60] and Singapore in collaboration with China [62]. No study was conducted in the Americas, in Africa or in Oceania. General and baseline characteristics are presented in Table 1.

Seven studies were clinical, comprising retrospective longitudinal or cross-sectional cohorts and, in two instances, analyses of open morphological databases [54,55,56,57,60,63,65]. Together, these contributed 1965 aneurysms to model development and internal validation, with a further 81 aneurysms in independent external cohorts. Four of the seven reported the number of patients as well as the number of aneurysms, totaling 848 patients [54,57,60,65]; the remaining three reported aneurysms only [55,56,63]. Age and sex were reported in four studies and were absent or incomplete in three, and no clinical study reported comorbidity, smoking status or family history in a form that would allow adjustment. Five studies were computational methodological studies without a clinical cohort [58,59,61,62,64]. These ranged from a single aneurysm geometry used as one of three test cases in a fluid dynamics methods paper [64], through corpora of 101 and 105 patient-derived geometries with high-fidelity simulations [59,61], to a parametric dataset of 984 synthetic middle cerebral artery bifurcation aneurysms [62] and a set of public datasets comprising 331 aneurysm and 1694 vessel surfaces [58].

Imaging provenance reflected this split. Among clinical studies, 3DRA or rotational DSA was the source in four [55,56,60,63], CTA in two [57,65], and a mixture of CTA and MRA in one [54]. The computational studies worked on meshes derived from public MRA-based datasets or on parametrically generated surfaces. Aneurysm location was tightly constrained in several cohorts: two studies included only internal carotid artery aneurysms [56,60], two only middle cerebral artery aneurysms [62,65], and two of the surrogate studies used sidewall aneurysms on a single idealized parent artery of fixed 4 mm diameter [59,61]. Only two studies reported a location distribution spanning both the anterior and the posterior circulation [55,57], and only two reported the aneurysm size distribution in a form permitting comparison with published cohorts [54,57].

### 3.3. Model Architectures and Input Representations

Architecturally, the field divides cleanly. Seven studies used point cloud networks of the PointNet or PointNet++ family, either to encode aneurysm surface shape [54,55,57], to encode spatially resolved hemodynamic fields produced by an upstream CFD run [56,60,63], or to act as a direct surrogate for the flow solution [62]. Four studies used graph neural networks in the strict sense: a physics-constrained MeshGraphNet operating on the volumetric simulation mesh [59], a transformer-based graph neural network with adjacency-masked self-attention for coupled Multiphysics fields [61], a spectral domain graph convolutional network using Laplacian eigenvectors as modes [64], and a graph neural network augmented with transferred surface embeddings for time-resolved flow prediction [58]. One study combined a geometric deep learning branch with a three-dimensional convolutional and transformer branch operating on the perianeurysmal image volume, fusing the two representations before classification [65].

Input representations followed the same division. Studies concerned with clinical classification sampled the aneurysm surface, typically at 2048 or 8192 points, and in three cases augmented the coordinates with nodal values of WSS, OSI, pressure, velocity and time-averaged WSS taken from a preceding CFD simulation [56,60,63]. Studies concerned with surrogacy operated on the simulation mesh itself, with node counts of the order of 20,000 to 65,000 and included among the node features the velocity at the current and preceding timesteps, its temporal derivative, inflow information and node type [59,61]. Two design choices recurred and both proved consequential. The first is how much of the parent vessel to retain: preserving a segment of the inflow artery improved rupture classification in one cohort, raising the AUC from 0.81 to 0.85 [55], and improved growth prediction in another, raising accuracy from 0.77 to 0.82 [54], whereas in a third study, addressing post-embolization recanalization, including the parent artery worsened performance [60]. The second is the explicit encoding of proximity to the wall: adding a Euclidean distance to wall feature was the single largest contributor to surrogate accuracy in the study that ablated it, and its removal degraded normalized mean absolute error (NMAE) from 4.05% to 4.70% [62].

### 3.4. Geometric Encoding for Clinical Endpoints

Seven studies addressed a clinical endpoint. Five used rupture status at the time of imaging as a surrogate for rupture risk [55,56,57,63,65], one predicted future aneurysm growth on follow-up imaging [54], and one predicted post-embolization recanalization [60]. Outcomes, models and results are presented in Table 2.

The consistent finding across this group is that learned geometric representations outperform conventional morphological indices. In the growth prediction study, a PointNet++ model applied to the aneurysm dome together with the parent vasculature achieved an accuracy of 0.82 with an AUC of 0.795, against 0.68 for a random forest on handcrafted features, 0.64 for a multilayer perceptron and 0.63 for the best single morphological threshold, the non-sphericity index; sensitivity reached 0.96 at a specificity of 0.63, a profile the authors argued suits a follow up strategy in which missing a growing aneurysm is the costlier error [54]. In the rupture status studies, the same pattern recurs. Adding PointNet derived hemodynamic cloud features to scalar morphological and hemodynamic parameters raised the accuracy of a support vector machine from 0.860 to 0.926 and its AUC from 0.925 to 0.969 in one cohort of 148 aneurysms [56], and in a second cohort of 149 aneurysms the best configuration reached an accuracy of 0.908 with an AUC of 0.944, with the notable result that sixteen dimensional cloud features outperformed 1024 dimensional ones, which the authors attributed to reduced redundancy and less overfitting [63]. A fully automated framework combining nnU-Net vessel segmentation with two pretrained PointNet++ stages for centerline labeling and aneurysm isolation achieved an AUC of 0.851 on 500 aneurysms, against 0.700 for the PHASES score computed on the same cases, and analysis of feature attributions indicated that aneurysm shape features contributed most and vessel shape features least [57]. The most methodologically complete study in this group combined a geometric branch with a neighborhood aware image branch and reported an accuracy of 91.46% with an AUC of 94.59% on a balanced internal test set, outperforming every published comparator, and it went further than any other included study in testing clinical utility: in a reader study with two radiologists and two neurosurgeons, model assistance raised diagnostic accuracy by approximately seven percentage points and mean specificity from 73.17% to 80.49%, with the largest gains among the less experienced readers [65].

Two caveats attach to these figures. The first is that the near-perfect discrimination reported in the recanalization study, an AUC and area under the precision-recall curve of 1.000 on the training set, arose in a cohort with only eight recanalized aneurysms, two of which were in the test set, and is best read as evidence of overfitting rather than of performance [60]. The second, and more important, is the behavior of these models outside their development cohort, considered below.

### 3.5. Surrogate Modeling of the Hemodynamic Solution

Five studies used graph or point cloud architectures to reproduce the CFD solution itself rather than to classify a clinical label [58,59,61,62,64].

The most complete of these trained a physics-constrained MeshGraphNet on 105 patient-derived geometries with 80 timeframes each and released the resulting corpus as an open benchmark [59]. Four variants were compared, and the configuration combining a physics-based loss with explicit inflow and acceleration features achieved a single-step root mean square error (RMSE) of 0.85 mm/s and a fifty-step rollout RMSE of 7.58 mm/s, against 1.11 and 50.51 mm/s for the unconstrained baseline, so that the physics constraints improved rollout stability by more than sixfold. Regional analysis showed the expected gradient of difficulty, with rollout error of 11.87 mm/s in the aneurysmal bulge, 8.38 mm/s at the neck and 4.66 mm/s in the parent artery. Mass conservation was respected to within a mean inlet-to-outlet difference of 3.7%, compared with 2.3% for the reference CFD, and the error in time-averaged WSS remained below 10% across the test set. Inference was near-instantaneous against a reference solver runtime of approximately one hour per case, with training requiring approximately twenty hours on commodity graphics processing units, so that the investment is recovered after roughly twenty simulations.

A companion study extended the approach to coupled multiphysics, predicting velocity, platelet concentration and thrombus fraction with a transformer-based graph neural network trained on finite element simulations of flow, platelet transport and biochemical reaction [61]. Training one model per physical field rather than a single multitask model improved accuracy by at least 32% in aggregate RMSE relative to state-of-the-art comparators, reduced parameter count by 21%, ran 45% faster than the next fastest model, and predicted the timing of thrombus formation correctly, whereas the multitask configuration showed a slight delay. A third study developed a geometry-aware point cloud surrogate on 984 synthetic middle cerebral artery bifurcation aneurysms and reported peak systolic velocity accuracy of NMAE 4.05% and relative L2 error 19.2% over the whole domain, with WSS accuracy of NMAE 2.59%, at inference times of approximately 1.6 s for velocity and 0.3 s for WSS [62]. A fourth used a spectral graph convolutional network with a deep neural network modeling the temporal evolution of each Laplacian mode, and on the aneurysm test case reduced relative L2 error to between 0.065 and 0.095 across velocity components, pressure and concentration, against 0.085 to 0.133 for a plain deep neural network and 0.208 to 0.314 for dynamic mode decomposition [64]. The fifth demonstrated that surface embeddings learned by a generative model trained on half a million non-medical three-dimensional assets can be transferred to aneurysm analysis, improving classification, segmentation and, in the flow prediction task, reducing simulation RMSE by approximately 15% [58].

### 3.6. Generalization, External Validation and the Transfer Gap

The findings of this review are least reassuring in precisely the domain that matters most for translation. Studies evaluating external validation, transfer testing, or out-of-distribution generalization are summarized in Table 3.

Independent external validation in a genuinely separate clinical cohort was performed in three studies. In the first, a point cloud model trained on one open database and validated on another saw its AUC fall from 0.85 to 0.71 for the configuration retaining the inflow artery, and from 0.81 to 0.69 for the dome-only configuration, in both cases falling below the AUC of 0.82 achieved by the morphology-based LASSO model published by the original database authors [55]. The second study was validated across imaging modalities, from CTA to 3DRA, and retained an AUC of 0.805 against 0.851 internally, although the external set comprised only 24 aneurysms and the report contains a numerical inconsistency in their allocation [57]. The third was validated on 57 aneurysms from four additional hospitals with different scanners and entirely unseen patients, and performance was essentially preserved, with an accuracy of 85.96% and an AUC of 94.32% [65].

Among the surrogate studies, generalization was tested against unseen inflow conditions and unseen anatomy rather than against an external clinical cohort. Two studies reported robustness to unseen inflow profiles without fine-tuning [59,61], although in one of them all models degraded appreciably when the boundary conditions were modified, with aggregate velocity error rising fivefold in the multitask configuration [61]. The most explicit test of anatomical transfer was conducted on five non-idealized, patient-derived geometries evaluated zero-shot by a model trained on parametric shapes: NMAE rose from 4.05% to 19.1% and relative L2 error from 19.2% to 62.4% and leave-one-out fine-tuning on the four remaining cases recovered only part of the loss, to 10.8% and 37.0%, respectively [62]. A comparable pattern appeared in the benchmark study, where the spatial distribution of the predicted fields was reproduced on four zero-shot patient-specific geometries, but the magnitudes were discrepant in two of them [59].

No included study linked a graph-based hemodynamic surrogate to an observed rupture, growth or stability event. The two studies identified in this review, geometric encoding for clinical classification and graph-based surrogacy of CFD, have not yet met.

### 3.7. Computational Performance, Data and Code Availability

Where reported, the computational gain was substantial and consistent. Inference times were of the order of seconds or less against reference solver runtimes measured in hours [59,61,62]. The cost is displaced rather than eliminated: training consumed approximately twenty hours on dedicated hardware in the study that reported it, and the corpus of high-fidelity simulations required to train these models is itself expensive to generate [59]. One study quantified the overhead of its feature extraction step, which was approximately thirty times longer than the baseline and dominated by the rendering of 200 views per geometry [58].

Four studies made data or code publicly available. One released an open benchmark of 105 patient-derived geometries with paired CFD fields [59], one released its implementation in a public repository [65], and two built on existing public morphological or geometric databases [55,57]. The remaining studies used institutional datasets that are not available for reuse, which places a hard limit on independent replication and on the construction of shared benchmarks.

### 3.8. Translational Stage of the Included Studies

Applying the descriptive ladder defined in Section 2.7 to the extracted data gives a clear picture of where this literature currently stands. Five studies sit at stage 1, having reported internal validation only, whether by cross-validation or by a held-out split, with no independent cohort and no deliberate test of robustness [54,56,60,63,64]. Four studies reached stage 2, adding explicit evaluation under conditions withheld from training: unseen inflow profiles and zero-shot patient-specific geometries in one case [59], modified inflow waveforms and extended cardiac cycle counts in another [61], out-of-distribution non-idealized geometries with leave-one-out fine-tuning in a third [62], and unseen micro-aneurysms and multiple aneurysm cases in the fourth [58]. Two studies reached stage 3 through independent external validation, one across curated open databases [55] and one across imaging modalities [57]. One study reached stage 4, combining multicenter external validation with a formal reader study in which model assistance altered clinician performance [65].

The distribution is informative in two ways. It confirms that the field has passed the proof-of-concept phase, since every included study reported quantitative performance against a defined reference rather than qualitative agreement. It also shows that the density of evidence falls sharply with each step towards the clinic, and that the two research programs described above occupy different rungs: the studies that reached stages 3 and 4 are all classification studies working on clinical cohorts, whereas every surrogate study of the hemodynamic solution remains at stage 1 or stage 2, tested against simulated rather than clinical references.

### 3.9. Risk of Bias and Reporting Completeness

Risk of bias was judged high or unclear in the analysis domain for the majority of included studies, driven by small development samples relative to the number of candidate predictors, class imbalance handled inconsistently, absence of calibration assessment in every study reporting a probabilistic classifier, and the use of rupture status at the time of imaging as a surrogate for prospective rupture risk. The participants domain was affected by narrow anatomical inclusion, by selection bias inherent to cohorts assembled from 3DRA, and by the absence of demographic and clinical covariates in several reports. In the surrogate studies, the reference standard domain was the dominant source of concern: each study generated its own ground truth, and the simplifying assumptions adopted, rigid walls, Newtonian rheology, generic inflow waveforms and, in one case, two-dimensional simulation, differed between studies and were not always accompanied by sensitivity analysis. Reporting completeness against TRIPOD+AI was variable, with the poorest coverage in the items concerning sample size justification, handling of missing data, calibration, and prespecification of the analysis. Full domain-level judgements are presented in Supplementary Table S3.

### 3.10. CFD and Multiphysics Simulation Assumption

Eight of the total twelve included studies developed on the themes of computational fluid dynamics (CFD) or multiphysics simulation, either as the source of the input features supplied to the network [56,60,63], as the ground truth the network was trained to reproduce [59,61,62], or as an externally generated flow dataset on which the architecture was based on [58,64]. In contrast, the remaining four studies operated on geometry or imaging alone and required no simulation [54,55,57,65]. In this context, the simulation set-ups of the eight studies are compared in Table 4, and they are heterogeneous in most extracted dimentions.

## 4. Discussion

This review identified twelve studies in which graph-based or geometric deep learning was applied to intracranial aneurysm geometry, summarized in Table 1 and Table 2, and four findings emerge from them. First, learned geometric representations of the aneurysm and of its parent vessel encode information about clinical behavior that conventional morphological indices do not capture, and the resulting classifiers outperform both handcrafted feature models and the PHASES score in internal testing. Second, graph and point cloud surrogates reproduce high-fidelity hemodynamic fields with errors in the range of a few per cent and with inference measured in seconds rather than hours, which is the order of improvement required to make hemodynamic biomarkers computable within a clinical encounter and, by extension, to make the flow environment created by an implanted device something that can be examined rather than assumed. Third, one of these models has already been extended from flow alone to the coupled problem of flow, platelet transport, and thrombus formation over a reactive surface, which is the computational representation of blood and biomaterial interaction. Fourth, and against all of this, performance degrades when the model meets anatomy, imaging or boundary conditions it has not seen; no model has been trained on a device-laden geometry, and not one study has connected a graph-based hemodynamic estimate to an observed clinical event.

### 4.1. Interpretative Framework: From Hemodynamics to Tissue-Level and Biomaterial Processes

This section will discuss the implications of these findings for biomaterials, tissue engineering, and neurovascular implant design as prospective applications rather than effects directly demonstrated by the included studies. From this perspective, the aneurysm is a mechanical problem only in part. What ruptures is a wall that has lost its endothelial lining, its smooth muscle cellularity and much of its organized collagen, and the transition from a stable to a degenerated wall is an inflammatory and matrix remodeling process rather than a purely structural one [13]. Hemodynamics enters that process as its boundary condition. Wall shear stress is the dominant mechanical stimulus sensed by the endothelium and it determines endothelial phenotype, junctional integrity and the inflammatory program downstream of it [12]; in resected human aneurysm domes, the flow conditions reconstructed from preoperative imaging correspond to the histological grade of wall degeneration and inflammatory infiltration [14]. A model that returns the intra-aneurysmal flow field in seconds is therefore not only accelerating a fluid calculation, but also making computable, at the bedside, the mechanical input to a tissue-level process that until now could only be inferred from the resected specimen.

The therapeutic side of the problem is explicitly a biomaterial one. Coil embolization, flow diversion and intrasaccular flow disruption do not repair the aneurysm wall; they install a scaffold that reshapes the flow so that the sac thromboses, and along which endothelial cells migrate to lay down a neointima that excludes the aneurysm from the circulation [8,9]. Occlusion is, in that sense, a tissue engineering outcome obtained with an implanted device, and its two determinants are the hemodynamic environment the device creates and the surface that the healing tissue must colonize.

### 4.2. What the Included Studies Contribute to the Biomaterials Question

Read against this framework, the twelve included studies fall into four groups whose relevance to implant design and evaluation is unequal but, in each case, definable.

The first group treats geometry as a proxy for wall biology. Bizjak and colleagues predicted future aneurysm growth in 44 aneurysms followed with CTA and MRA and found that deep shape features extracted by PointNet++ from the surface mesh reached an accuracy of 0.82 with a sensitivity of 0.96, against 0.68 for a random forest on handcrafted indices and 0.63 for the best single morphological threshold [54]. Growth is the clinical surrogate of progressive wall degeneration, so this is, in effect, a demonstration that the shape of the sac carries a readable signature of the remodeling state of its wall, which is the tissue-level variable that determines whether a lesion needs a device at all. Cao and colleagues reached the same conclusion from a different direction in 623 aneurysms drawn from the AneuX databases, where retaining a segment of the inflow artery alongside the dome raised the area under the curve (AUC) from 0.81 to 0.85 [55]; the parent vessel matters because it defines the inflow jet, and the inflow jet is what a flow diverter is deployed to redirect. Choi and colleagues built the most complete automated pipeline in this group, segmenting the vasculature with nnU-Net and isolating the aneurysm with two pretrained PointNet++ stages before classification, and obtained an AUC of 0.851 in 500 aneurysms against 0.700 for PHASES, with feature attribution showing that aneurysm shape features contributed most and vessel shape features least [57].

The second group makes the hemodynamic field itself the input to a clinical classifier and is therefore the closest existing analog of what device planning would require. Chen and colleagues computed patient-specific CFD in 148 internal carotid artery aneurysms and used a PointNet to extract hemodynamic cloud features from the impingement zone and inflow jet, raising the accuracy of a support vector machine from 0.860 to 0.926 and its AUC from 0.925 to 0.969 [56]; the impingement zone is where the wall is thinnest and where a coil mass or a diverter must alter the flow. Shou and colleagues repeated the design in 149 aneurysms and found that sixteen-dimensional cloud features outperformed 1024-dimensional ones, reaching an accuracy of 0.908 and an AUC of 0.944, which suggests that the useful hemodynamic signal is compact and that redundancy actively harms these models [63]. Liao and colleagues addressed the failure mode of the implanted construct directly, predicting post-embolization recanalization in 52 internal carotid and posterior communicating artery aneurysms; their central result is that a PointNet fed with three-dimensional velocity fields outperformed the same architecture fed with morphology alone by 21.3 percentage points of AUC, and outperformed machine learning models built on spatiotemporally averaged scalars including the volume embolization ratio [60]. That is a direct statement that packing density and other zero-dimensional summaries discard the information that decides whether a coiled aneurysm stays occluded. Zhou and colleagues produced the most clinically mature study in the review, fusing a geometric branch on the aneurysm and parent artery with a neighborhood aware branch on a cropped CTA volume, reaching an accuracy of 91.46% and an AUC of 94.59% internally, 85.96% and 94.32% across four external hospitals, and, uniquely, showing in a reader study that model assistance raised clinician accuracy by approximately seven percentage points and mean specificity from 73.17% to 80.49% [65].

The third group learns the physics rather than the label, and it is where the computational argument for these architectures is strongest. Lannelongue and colleagues trained a physics constrained MeshGraphNet on 105 patient-derived geometries with 80 timeframes each and released the corpus as an open benchmark; the variant combining a physics loss with inflow and acceleration features reduced fifty step rollout error from 50.51 to 7.58 mm/s, respected mass conservation to within 3.7% against 2.3% for the reference CFD, and kept the error in time averaged wall shear stress below 10% across the test set, with regional error rising predictably from the parent artery, 4.66 mm/s, through the neck, 8.38 mm/s, to the bulge, 11.87 mm/s [59]. That regional gradient matters for device work, because the neck is exactly where a diverter must act and where the surrogate is least accurate. Sheng and colleagues built a geometry-aware point cloud surrogate on 984 parametric middle cerebral artery bifurcation aneurysms and predicted peak systolic velocity to a normalized mean absolute error of 4.05% and wall shear stress to 2.59%, in approximately 1.6 and 0.3 s per case, with the distance-to-wall feature the dominant contributor [62]; a parametric dataset of this kind is precisely the substrate on which a device design space could be explored. Wen and colleagues approached the same problem from numerical analysis, using Laplacian eigenvectors as modes in a spectral graph convolutional network and reducing relative L2 error on the aneurysm test case to between 0.065 and 0.095, against 0.208 to 0.314 for dynamic mode decomposition [64]. Hervé and colleagues showed that surface embeddings learned by a generative model trained on half a million non-medical three-dimensional objects transfer to aneurysm analysis, improving classification, segmentation and, in the flow task, reducing simulation error by approximately 15% [58]; for a field whose central constraint is the scarcity of annotated vascular geometries, and which would face that constraint again when assembling device-laden datasets, cross-domain transfer is a strategically important result.

The fourth group consists of a single study, and it is the one that speaks most directly to the theme of this Special Issue. Pelissier and colleagues trained a transformer-based graph neural network with adjacency-masked self-attention on coupled multiphysics simulations of flow, platelet transport and biochemical reaction, and predicted thrombus formation inside patient-specific aneurysm geometries containing a reactive surface [61]. Training one model per physical field rather than a single multitask model improved aggregate error by at least 32% against state-of-the-art comparators, reduced parameter count by 21%, ran 45% faster than the next fastest model, and reproduced not only the extent of the clot but the timing of its formation, whereas the multitask configuration lagged. Stripped of its engineering vocabulary, this is an in silico model of the blood and material interaction that decides whether an embolized aneurysm occludes, and it is the first demonstration that such a model can be learned rather than solved. Its limitations are equally instructive: the simulations were two-dimensional, the inflow profiles were generic rather than patient-specific, and the reactive surface was randomized rather than configured to represent a particular device.

### 4.3. Future Perspective: Potential Implications for Implant Design and Evaluation

The clinical case for these models does not rest on accuracy alone but on what accuracy at speed could make possible, with these applications representing prospective directions interpreted from the included studies’ conclusions. At present, testing one flow diverter configuration in one patient-specific anatomy costs hours of solver time, which confines virtual treatment planning to a handful of scenarios chosen in advance. The benchmark study included here made the arithmetic explicit: approximately twenty hours of training against approximately one hour per simulation, with the investment recovered after roughly twenty cases [59]. At seconds per evaluation, the porosity, pore density, landing zone and degree of oversizing of a braided implant, or the packing density and spatial distribution of a coil mass, become parameters over which an optimization can run rather than variables chosen by experience. A reinforcement learning study of patient-specific stenting of intracranial aneurysms anticipated exactly this workflow [66], and its limiting factor was the cost of the underlying solver, which is the cost these surrogates remove. The same argument scales beyond the individual patient. Regulatory science is moving towards accepting computational evidence as part of device evaluation, and in silico trials require populations of virtual patients large enough to be statistically meaningful, which is unattainable with conventional finite element and finite volume solvers and becomes plausible with validated surrogates [67]. The digital twin concept, already articulated for cardiac care, presupposes exactly this combination of patient-specific geometry and near-instantaneous physics [68].

### 4.4. Device-Laden Anatomy and Neurovascular Biomaterials

The most visible gap in the biomaterials theme is that none of the included models were trained on device-laden aneurysm anatomy. This is important because the setting in which these tools would support the follow-up of implant development is the treated aneurysm, where coils, flow diverters or intrasaccular devices reshape the lumen and impose new boundary conditions. A clinically useful device-specific surrogate would therefore need to represent much more complex structures and interactions, including porous-media or resolved-strut effects across braided implants, local flow disruption produced by coil mass distribution, fluid–structure interactions between blood, vessel wall and implanted material, and, finally, material-dependent surface chemistry influencing thrombus formation and endothelial coverage.

Among the included articles, the post-embolization recanalization study showed that three-dimensional velocity-field features outperformed morphology alone, supporting the relevance of spatial hemodynamic information after treatment [60]. In this scenario, the most promising model for a blood–material interaction framework was the transformer-based graph network by Pelissier et al., which learned coupled simulations of flow, platelet transport and thrombus formation over a reactive surface [61]. Despite this advance, the model remained a two-dimensional proof of principle with generic inflow conditions and a randomized, non-device-specific reactive surface. Thus, future work should extend graph and geometric deep learning to explicit implant architecture, and porous-media or fluid–structure interactions, enabling the creation of new parameters and interpretations for these advanced geometries.

### 4.5. Why Graph and Point Set Architectures, and Where They Still Struggle

The architectural argument is not merely fashionable. Convolutional networks assume a regular grid, and imposing one on a cerebral aneurysm requires interpolating an unstructured tetrahedral mesh onto a voxel lattice, which blurs the near-wall region where wall shear stress is defined and discards the local connectivity that determines whether two mesh points are hydrodynamically adjacent or separated by a wall. This limitation would be aggravated, not relieved, by the presence of a braided implant, whose struts are subvoxel structures. Graph neural networks pass messages along the edges of the simulation mesh itself, so adjacency is respected by construction, arbitrary resolution is tolerated, and the vortical and helical structures characteristic of intra-aneurysmal flow are representable [27,32,33]. Point cloud networks achieve permutation-invariant encoding of unordered surface samples, which suits geometry that has no canonical parameterization [30,31]. The included studies bear this out in direct comparison, as set out in Table 2: a spectral graph convolutional network outperformed dynamic mode decomposition and a plain deep neural network on aneurysmal flow by a wide margin [64], and point cloud hemodynamic features improved rupture classification over the scalar summaries of the very simulations from which they were drawn [56,63]. In the coronary circulation, a gauge equivariant mesh convolutional network reproduced CFD-derived wall shear stress closely enough to preserve its association with a clinical endpoint [37].

Three limitations of the paradigm recur across the included studies and deserve to be stated plainly. The first is data hunger. These models require corpora of high-fidelity simulations that are costly to assemble, and the largest patient-derived corpus identified here contains 105 geometries [59]. The second is the message passing bottleneck: inflow information must traverse many successive rounds of propagation to reach distal mesh regions, which forces greater network depth and lengthens training. The transformer-based and multi-resolution variants in this review respond directly to that problem [58,61]. The third, and the most consequential clinically, is out-of-distribution fragility. In the most explicit test, normalized error rose from 4.05% to 19.1% when a model trained on parametric geometries met patient-derived anatomy, and leave-one-out fine-tuning recovered only part of that deficit, to 10.8% [62]. The benchmark study showed the same pattern more weakly: spatial patterns transferred to four zero-shot patient-specific cases, but magnitudes were discrepant in two [59]. Two of the surrogate studies were trained on semi-idealized sidewall aneurysms sharing a single parent artery of fixed 4 mm diameter [59,61], which is defensible for isolating the physics but a poor proxy for the anatomical variability a clinical or a device evaluation model would encounter.

The physics-informed and physics-constrained approach is the most promising route out of the first and third of these difficulties, since embedding residuals of the governing equations in the loss reduces dependence on labeled simulation data and constrains the solution space to physically admissible fields [35]. The benchmark study provides direct evidence of the benefit, the physics-constrained variant having improved rollout error more than sixfold over the unconstrained baseline [59]. Cross-domain transfer offers a complementary route, whose main cost at present is the computational overhead of the encoding step, approximately thirty times the baseline and dominated by the rendering of 200 views per geometry [58].

### 4.6. The Gap Between Discrimination and Clinical Usefulness

The classification studies present the familiar problems of prediction model research rather than anything peculiar to graph architectures. Five of the seven clinical studies used rupture status at the time of imaging as the outcome [55,56,57,63,65]. This is a cross-sectional contrast between aneurysms that have bled and aneurysms that have not, and it is not the prospective probability that an unruptured aneurysm will bleed; ruptured aneurysms are imaged after the event, their geometry may have been altered by the rupture, and the case mix of a ruptured cohort differs systematically from the population in which a clinician actually needs a prediction. The two studies with a genuinely prospective endpoint, future growth [54] and post-embolization recanalization [60], are the more clinically faithful designs, and both were conducted in cohorts of fewer than sixty aneurysms.

Additionally, none of the clinical studies reported calibration, limiting the interpretation of predicted probabilities. A model may achieve high discrimination while systematically overestimating or underestimating absolute risk; consequently, it is imperative that future studies should assess and report calibration alongside discrimination, particularly when models are intended to assess individualized risk estimation and clinical decision-making [69]. Systematic appraisals of machine learning prediction models in other clinical domains have repeatedly documented this combination of high reported discrimination, absent calibration, small samples and optimistic internal validation, together with a strong tendency to overinterpret the resulting figures [70,71,72], and the studies in this review are not exceptions. The recanalization study reporting an AUC of 1.000 in a cohort containing eight events is the clearest illustration [60]. External validation, where performed, was informative in both directions: the fall from an AUC of 0.85 to 0.71 when a point cloud model crossed from one curated open database to another, dropping below the morphological model it was designed to beat, quantifies the cost of cohort-specific selection bias [55], whereas the study that validated on 57 aneurysms from four hospitals with different scanners and preserved an AUC above 0.94 shows that robust transfer is attainable [65].

### 4.7. From Experimental Innovation to Clinical Translation

Placing the twelve studies on the descriptive ladder defined in Section 2.7 makes the shape of the problem visible. Every study has cleared the stage of proof of concept, but five remain at internal validation alone [54,56,60,63,64], four have added deliberate robustness testing [58,59,61,62], two have achieved independent external validation [55,57], and one has demonstrated an effect on clinician performance [65]. The two research programs occupy different rungs, and they do so systematically: the studies that reached external validation and clinical utility are all classification studies working on clinical cohorts, whereas every surrogate of the hemodynamic solution remains at stage 1 or stage 2, validated against a simulation rather than against a patient.

That separation is the central obstacle to the translational agenda this Special Issue addresses. The engineering groups building graph surrogates validate against the solver and report physical error metrics; the clinical groups building geometric classifiers validate against rupture status and report areas under the curve. Neither has taken the step that would join them, which is to use a surrogate-derived hemodynamic field as the input to a clinical or device-related prediction and to test the resulting estimate against an observed event. The only demonstration of this kind in any vascular bed is in the coronary circulation, where a gauge-equivariant mesh network preserved the association between wall shear stress and myocardial infarction that CFD-derived wall shear stress provides [37]. Three of the classification studies in this review already computed patient-specific CFD to extract their hemodynamic features [56,60,63], so the design exists in embryo; an accurate surrogate would simply remove the step that makes such studies prohibitive at scale.

Three further obstacles stand between the present models and their use in implant design, and they should be stated plainly rather than left implicit. First, every surrogate identified in this review was trained on untreated aneurysms. A deployed device alters the geometry, introduces a porous or braided internal boundary, and couples the fluid to a compliant solid structure, none of which appears in the training corpora described in Table 1; extending these models to device-laden anatomy requires simulation datasets that include the implant and that do not currently exist in the public domain. Second, all reference simulations assumed rigid walls and Newtonian rheology wherever the rheological model was specified, which excludes the wall mechanics that determine whether an aneurysm remodels or ruptures and that any serious model of the tissue response would have to include. Third, the biological half of the process is slower and less amenable to continuum description than the flow: thrombus organization and endothelialization unfold over weeks to months and depend on surface chemistry, coating and cell–substrate interaction as much as on shear, and they were represented in only one of the included studies, in two dimensions and with a randomized rather than a device-specific reactive surface [61]. Closing that gap is where the interests of the computational and the biomaterials communities coincide most directly.

In the context of translational readiness, a significant difference was noticed between classification and hemodynamic surrogate studies. Five of the twelve studies did not go past Stage 1, while all studies reaching external validation or clinical utility were classification models. In this scenario, Zhou et al. [65] was the only study to reach Stage 4, demonstrating improved clinician performance in a reader study. In contrast, all hemodynamic surrogates remained at Stages 1–2, demonstrating computational acceleration, agreement with CFD, or robustness testing, but no external clinical development. In conclusion, the classification models shifted along the translational pathway, whereas hemodynamic surrogates were primarily directed at the computational and engineering validation stage.

A related boundary question deserves comment. Work that constructs an explicit semantic vessel graph from a deep learning segmentation and then analyzes its curvature and torsion in relation to rupture status sits close to this review without falling inside it, because the graph is a representation rather than a learned predictor [73]. As graph representations of the cerebral vasculature become more common, the distinction between using a graph and learning on a graph will need to be drawn carefully in future syntheses.

### 4.8. Strengths and Limitations of This Review

The review has several strengths. The search covered four databases with strategies peer-reviewed against the PRESS checklist and reported in full, supplemented by citation tracking, preprint and conference searching and inspection of dataset repositories, which matters unusually in a field whose vocabulary is poorly represented in MeSH and Emtree. Screening, full-text assessment and extraction were performed in duplicate. Extraction included the solver configuration and physical assumptions underlying each reference simulation, an item routinely omitted but which determines the meaning of every reported accuracy figure. Risk of bias was appraised with an instrument designed for prediction models rather than for clinical trials, supplemented by artificial intelligence-specific signaling items, and reference standard heterogeneity was handled as a distinct domain.

The limitations are equally clear. The evidence base is small, and twelve studies of markedly heterogeneous design do not support quantitative pooling; accuracy was reported on non-commensurable scales, against reference simulations that differ between studies, and for endpoints ranging from a continuous physical field to a binary clinical label, so a pooled summary estimate would have no interpretable meaning. Our synthesis is therefore narrative and tabular, and comparisons across studies are indicative rather than formal. The literature is dominated by a small number of groups, with three of the five surrogate studies from a single center, so architectural preferences and reference assumptions are correlated with authorship. Moreover, most clinical studies addressed cross-sectional rupture status rather than prospective rupture risk, limiting interpretation of their discrimination as evidence of future-event prediction. Publication and reporting bias cannot be assessed with the usual tools in a field of this size, and since methodological papers reporting negative transfer are unlikely to be written, the true generalization gap is probably wider than the one we observed. More than half of the included reports appeared within eighteen months of the search, so any synthesis of this topic dates quickly, and a top-up search was performed immediately before submission. Finally, several included reports contained internal numerical inconsistencies, which we recorded rather than resolved, and in two cases these affect the exact composition of the validation sets. The translational stage classification is descriptive, was applied post hoc, and should be read as a way of organizing the evidence rather than as a validated instrument.

## 5. Conclusions

Graph and geometric deep learning has reached the point where the intra-aneurysmal flow field can be reproduced in seconds, with errors of a few per cent against the high-fidelity simulation it replaces, where learned representations of aneurysm shape discriminate rupture, growth and post-embolization recanalization better than the morphological indices in current use, and where the coupled problem of flow, platelet transport and thrombus formation over a reactive surface has been learned rather than solved. That is a genuine advance and substantially reduces the computational burden that has limited hemodynamic analysis in neurovascular practice that existed for two decades. It also brings within reach something the biomaterials community has wanted for longer, namely the ability to evaluate the flow environment created by a coil mass or a braided implant, and therefore the thrombotic and endothelial response on which durable occlusion depends, across a design space rather than in a single simulation.

What these models do not yet do is generalize reliably to anatomy they have not seen, and none has been tested against a prospective clinical event or trained on a geometry containing a device. The field is accordingly at the stage where methodological ingenuity is no longer the limiting factor. Progress now depends on shared benchmarks built from patient-specific geometries with documented solver assumptions, on prespecified external validation as a condition of publication rather than an optional extra, on the reporting of calibration alongside discrimination, on the extension of training corpora to implanted anatomy, and above all on studies in which a surrogate derived hemodynamic estimate is carried through to an observed outcome in a followed cohort. Until that work is done, these models remain an elegant solution to the cost of computing hemodynamics rather than to the clinical problem of deciding which aneurysm to treat, and with what.

## Figures and Tables

**Table 1:** General and baseline characteristics of the twelve included studies.

Study	Country	Design	Imaging	Patients/Aneurysms (n)	Comparison Groups (n)	Age (Years)	Female, n (%)	Aneurysm Location	Aneurysm Size	Input Features	Model(s)	Validation	Primary Endpoint	Clinical vs. Synthetic
Bizjak 2021 [54]	Slovenia (Ljubljana)	Retrospective longitudinal cohort, single center	CTA (20), MRA (24)	39/44	Growing 25 (57%) vs. stable 19 (43%); expert-adjudicated	Range 43–85; median 67	24 (61.5)	PComA 25; ophthalmic 13; superior hypophyseal 6	Median 5.01 mm (1.4–12.2); <3.9 mm n = 11, 4–10 mm n = 30, >10 mm n = 3	Five conventional morphologic features plus deep shape features from surface meshes	Univariate thresholds; random forest; MLP; PointNet++	Four-fold cross-validation	Future aneurysm growth	Clinical
Cao 2024 [55]	China (Guangzhou); European cohorts	Retrospective modeling study on open databases (AneuX)	3DRA	NR/623	Ruptured 211 vs. unruptured 412 (HUG 124/340; @neurIST 87/72)	Mean ± SD: R 53.4 ± 13.3; U 55.2 ± 12.4	R 143 (67.8); U 323 (78.4)	R vs. U—ICA 26/156; MCA 33/126; PComA 63/44; AComA 55/41; posterior 23/30; ACA 11/15	NR	Aneurysm dome/cut1 point clouds	PointNet++; LASSO comparator	HUG training/internal validation (464); @neurIST external validation (159)	Rupture status	Clinical database
Chen 2022 [56]	China (Beijing)	Retrospective cross-sectional study with patient-specific CFD, single center	3DRA/rotational DSA	NR/148	Ruptured 39 vs. unruptured 109	NR	NR	100% ICA; sidewall vs. bifurcation	Not reported as strata; 10 morphological variables measured	Morphologic variables; CFD hemodynamic parameters; PointNet-derived hemodynamic cloud features	RF; KNN; XGBoost; SVM; LightGBM + PointNet feature extractor	Training/testing procedure reported; no external cohort	Rupture status	Clinical + CFD
Choi 2025 [57]	South Korea (Seoul); Slovenia	Retrospective single-center cohort with external validation	CTA; 3D-RA (external)	335/500 (+24 external)	Ruptured 250 vs. unruptured 250; external 10 vs. 15 (sums to 25 vs. “24 IAs”—paper inconsistency)	Median 54 (IQR 45–67)	222 (66.2)	MCA 119 (23.8); AComA 118 (23.6); PComA 97 (19.4); ant. choroidal 26 (5.2); basilar apex 24 (4.8); others 87 (17.4)	Median max diameter 6.78 mm; <5 mm 144 (29%), 5 ≤ 10 mm 288 (58%), ≥10 mm 68 (13%)	Automated vessel/aneurysm shape embeddings + 20 conventional features	nnU-Net; PointNet++; RF/XGB/MLP/LR/SVM/NB/KNN/DT	Five-fold cross-validation + Aneurisk external validation	Rupture status	Clinical
Hervé 2026 [58]	France (Mines Paris)	Computational methodological study (public-benchmark)	3D meshes/point clouds (MRA-derived)	NA/331 IAs + 1694 vessels; 116 annotated; 101 AnXplore models	Aneurysm vs. healthy vessel (classification); aneurysm vs. vessel region (segmentation); flow fields (regression)	NA	NA	Not specified in the public datasets	Not reported per case; includes micro-aneurysms and multiple-aneurysm cases	TRELLIS embeddings and conventional surface descriptors	TRELLIS-enhanced classifiers; PointNet/PointNet++ comparisons	Held-out classification experiments	Aneurysm analysis/classification performance	Clinical imaging-derived surfaces
Lannelongue 2026 [59]	France (Mines Paris; Nice)	Computational methodological study; release of the BenchAnXplore benchmark	3D meshes; high-fidelity CFD ground truth	NA/105 (80 timeframes each) + 4 OOD	Train 95 vs. test 10; plus 6 unseen inflow profiles and 4 zero-shot patient-specific cases	NA	NA	Sidewall aneurysms on the internal carotid artery (siphon-like curvature)	Bulges 3–9 mm; parent-vessel diameter fixed at 4 mm	Graph geometry, boundary conditions and physics constraints	Physics-constrained graph neural network	Benchmark train/validation/test split and generalization experiments	Hemodynamic field prediction	Patient-derived geometry + CFD
Liao 2023 [60]	Japan (Kanazawa)	Retrospective consecutive-patient cohort with CFD, single center	3D-RA pre-treatment; MRA follow-up	51/52	Recanalized 8 vs. stable 44 (Table 1 of the paper lists 45—inconsistency)	Mean ± SD: 65 ± 13 vs. 69 ± 10 (*p* = 0.456)	6 vs. 38	100% ICA–posterior communicating (ICPC)	Max size, height and neck width significantly larger in recanalized (*p* < 0.05); dimensionless indices NS	3D morphology and CFD velocity/hemodynamic fields	Velocity-PointNet; conventional PointNet; comparator ML models	Cross-validation/resampling reported in article	Post-embolization recanalization	Clinical + CFD
Pelissier 2026 [61]	France (Mines Paris)	Computational methodological study (multiphysics surrogate)	IntrA-derived geometries; multiphysics CFD ground truth	NA/101	Train 80 vs. validation 11 vs. test 10	NA	NA	Sidewall aneurysms near the ICA siphon	Parent-vessel diameter 4 mm; 0.05 mm mesh; ~65,000 nodes per geometry	Unstructured mesh, flow, platelet transport and biochemical fields	Transformer-based graph neural networks; multitask and field-specific models	80/11/10 case split; unseen inflow and cardiac-cycle robustness tests	Spatiotemporal thrombosis/physical-field prediction	Patient-specific geometry + simulation
Sheng 2026 [62]	Singapore; China	Computational methodological study on a synthetic parametric dataset	None (parametric geometry); CFD ground truth	NA/984 (+5)	Train: validation: test = 8:1:1; 5 non-idealized cases zero-shot and LOOCV	NA	NA	MCA bifurcation apex, saccular	Sampled: W 4.0–6.0 mm; H 5.0–9.0 mm; D1 2.2–3.0 mm; D2 1.6–2.2 mm; D3 1.8–2.6 mm; angles 0–40°	Point cloud plus distance-to-wall feature	Geometry-aware single-input PointNet surrogate	Held-out test set + out-of-distribution non-idealized geometries	Velocity field and WSS prediction	Synthetic
Shou 2024 [63]	China (Beijing)	Retrospective cross-sectional study with patient-specific CFD, single center	3D rotational DSA	NR/149	Ruptured 40 vs. unruptured 109	NR	NR	Sidewall vs. bifurcation; daughter sacs recorded; specific site not detailed	Not reported as strata; geometric parameters measured in Geomagic	Geometric parameters; CFD hemodynamic parameters; 16/32/64/1024-dimensional PointNet cloud features	XGBoost; CatBoost; SVM; LightGBM; logistic regression	Repeated training/testing described in article	Rupture status	Clinical + CFD
Wen 2023 [64]	China (Beijing)	Fluid-dynamics methodological study (non-clinical); the aneurysm is one of three test cases	None (CFD simulation data)	NA/1 geometry (ICA region), Re ≈ 100	200 training vs. 100 test snapshots (non-overlapping)	NA	NA	Internal carotid artery region	NA	Graph-structured flow snapshots on unstructured grids	Spectral graph convolutional DNN; DMD; DNN	Held-out temporal snapshots/benchmark comparisons	Velocity, pressure and related flow-field prediction	Computational benchmark
Zhou 2025 [65]	China (Wenzhou; Shanghai)	Retrospective single-center cohort with multicenter external test and reader study	3D CTA; rupture confirmed by SAH + DSA/CTA/surgery	423/449 (+57 external)	Ruptured 308 vs. unruptured 141; balanced test 41 + 41; imbalanced 208/41; external 37/20	NR	NR	100% middle cerebral artery	IAs < 3 mm excluded	3D geometric branch + neighborhood-aware 3D CNN/Transformer branch	GN-Net; conventional ML and deep-learning comparators	Balanced and imbalanced internal tests; external set from four hospitals	Rupture status	Clinical

NR, not reported; NA, not applicable. Additionally: 3DRA/3D-RA, three-dimensional rotational angiography; ACA, anterior cerebral artery; AComA, anterior communicating artery; CFD, computational fluid dynamics; CNN, convolutional neural network; CTA, computed tomography angiography; DSA, digital subtraction angiography; IA, intracranial aneurysm; ICA, internal carotid artery; ICPC, internal carotid-posterior communicating artery; IQR, interquartile range; KNN, k-nearest neighbor; LOOCV, leave-one-out cross-validation; LR, logistic regression; MAE, mean absolute error; MCA, middle cerebral artery; ML, machine learning; MLP, multilayer perceptron; MRA, magnetic resonance angiography; NS, not significant; OOD, out-of-distribution; PComA, posterior communicating artery; RF, random forest; SAH, subarachnoid hemorrhage; SD, standard deviation; SVM, support vector machine; WSS, wall shear stress.

**Table 2:** Outcomes, geometric deep-learning models and results of the twelve included studies.

Study	Outcome	Reference Standard	Geometric DL Model	Inputs	Comparators	Validation	Best-Model Performance	External Validation	Key Finding	Main Limitations
Bizjak 2021 [54]	Future aneurysm growth (growing vs. stable)	Side-by-side visual comparison of baseline and follow-up surface meshes by a neurosurgeon (>10 y experience)	PointNet++ (dome + parent vasculature; and dome only)	Surface mesh resampled to 2048 points + normals (6 × N). Handcrafted set: HMAX, volume, surface area, NSI, AR	Univariate thresholding (5 features), random forest, MLP	4-fold cross-validation (11 cases per fold), class ratio preserved	PointNet++ (dome + parent vessel): accuracy 0.82, AUC 0.795, sensitivity 0.96, specificity 0.63. Dome only: 0.77/0.72. RF 0.68/0.66; MLP 0.64/0.62; best univariate (NSI) 0.63/0.62. Correct in 37/44 IAs	None	Deep shape features clearly outperform conventional morphological indices; high sensitivity supports surgical prioritization and the “no-treatment” follow-up paradigm; including the parent vasculature improves performance	Small sample (44 IAs); moderate specificity (0.63); single-center, non-public dataset; mesh-extraction artifacts may raise false positives; no external validation
Cao 2024 [55]	Rupture status (surrogate for rupture risk)	Rupture status recorded in the HUG and @neurIST (AneuX) databases; indeterminate cases excluded	PointNet++ on point clouds, two clipping configurations: “dome” and “cut1” (dome + inflow artery)	3D point clouds, 8192 points by farthest point sampling	PCA-based LASSO regression published by the original database authors (internal AUC 0.82)	Stratified 5-fold cross-validation in HUG (4:1); independent external validation in @neurIST	Internal (HUG), 5-fold means: cut1 AUC 0.85/accuracy 0.82/sensitivity 0.72/specificity 0.86; dome 0.81/0.79/0.61/0.86	Yes—@neurIST (87 R/72 U): AUC falls from 0.85 to 0.71 (cut1) and 0.69 (dome), below the original LASSO-PCA (0.82)	Point clouds are a feasible representation for rupture-risk assessment, and preserving the inflow artery (cut1) beats the isolated dome; however, cross-cohort generalizability is a concrete concern	Marked heterogeneity in ruptured/unruptured composition between HUG and @neurIST; 3DRA-related selection bias; no clinical or hemodynamic covariates; rupture used as surrogate; no prospective multicenter validation
Chen 2022 [56]	Rupture status (ruptured vs. unruptured)	Clinical rupture status recorded at Beijing Tiantan Hospital	Integrated model: PointNet extracting hemodynamic point-cloud features (8 → 1024 dimensions) feeding machine-learning classifiers	Group A: 10 morphological variables + hemodynamic parameters. Group B: A + hemodynamic cloud features from 5000 points (WSS, OSI, pressure, velocity, TAWSS in the impingement zone and inflow jet)	Random forest, KNN, XGBoost, SVM, LightGBM—each with Group A vs. Group B	10-fold cross-validation repeated 100 times with random reshuffling	SVM with Group B: accuracy 0.926, AUC 0.969 (95% CI 0.967–0.970), sensitivity 0.850, specificity 0.954. Group B—RF 0.908/0.952; LightGBM 0.917/0.965; XGB 0.900/0.950. Group A—SVM 0.860/0.925	None	Hemodynamic cloud features significantly improve classification over scalar hemodynamic and morphological parameters (*p* < 0.05); impingement-zone and inflow-jet information is discriminative; SVM most robust	Small, imbalanced sample (148 IAs); ICA aneurysms only; non-patient-specific flow boundary conditions; impingement zone defined by an arbitrary threshold (>80% max WSS); no external validation
Choi 2025 [57]	Rupture status at imaging (surrogate for rupture risk)	Visual labeling of CTA by an experienced radiologist and neurosurgeon; ruptured IAs imaged on the day of the event	Fully automated three-stage DL framework: nnU-Net vessel segmentation → pretrained PointNet++ centerline labeling → pretrained PointNet++ aneurysm isolation	Vessel-shape latent features (1024), aneurysm-shape features (2048 points), 17 conventional morphological features, location, age, sex	PHASES score; 8 ML models (RF, XGB, MLP, LR, SVM and others)	Stratified 5-fold cross-validation (1:1 ruptured:unruptured per fold; age and sex distributions checked); ablation and feature selection	Random forest: AUC 0.851, accuracy 0.782, sensitivity 0.804, specificity 0.760—201/250 ruptured and 190/250 unruptured correctly identified. PHASES: AUC 0.700, accuracy 0.638 at threshold 8	Yes—Aneurisk 3D-RA (different modality): AUC 0.805	A fully automated framework built on screening scans outperforms PHASES and transfers across imaging modalities; SHAP shows aneurysm-shape features contribute most and vessel-shape features least	Rupture status at imaging is not prospective risk; key clinical variables unavailable (e.g., SAH history); small external set (24 IAs) with a numerical inconsistency in the report
Hervé 2026 [58]	Three technical tasks: aneurysm vs. vessel classification; aneurysm/vessel mesh segmentation; time-resolved blood-flow prediction	Reference labels and annotations from Intra3D/IntrA; AnXplore CFD simulations for flow	TRELLIS surface features (3D generative encoder pretrained on 500,000 non-medical assets) replacing point normals and mesh descriptors, feeding PointNet, PointNet++, MLP and logistic regression; GNN + Transformers for flow	Point clouds (512/1024/2048 samples) with positions and 1024-dimensional TRELLIS vectors; mean, SD, min and max features for segmentation	Baselines without TRELLIS (coordinates and normals); published Intra3D results (PointNet++, PointCNN, SO-Net) and 3DMedPT	Classification: 5 runs over 5 seeds. Segmentation: 5-fold cross-validation. AdamW; 100 epochs with TRELLIS vs. 300 without	Classification—PointNet++ + TRELLIS (512 pts): vessel accuracy 99.88%, aneurysm 100%, F1 0.999 (baseline 90.30%/52.59%/0.813; 3DMedPT 99.02%/94.06%/0.920). Segmentation—PointNet + TRELLIS (2048): IoU 96.57%/88.67%, DSC 98.26%/93.99%. Flow: ~15% RMSE reduction	Not performed in an independent clinical cohort; robustness shown for unseen micro-aneurysms and multiple-aneurysm cases	Cross-domain transfer of geometric features learned on non-medical 3D data substantially improves all three tasks and beats the state of the art, even with a plain MLP and no point-cloud-specific architecture	High computational cost of the rendering/encoding step (~30× longer; 200 Blender views ≈ 80% of runtime); no clinical or demographic data in the datasets used; no clinical external validation
Lannelongue 2026 [59]	Time-resolved 3D hemodynamic fields across the cardiac cycle (velocity, WSS, TAWSS) as a CFD surrogate	High-fidelity CFD simulations over the 105 BenchAnXplore geometries; 80 timeframes per case	Physics-constrained MeshGraphNet—four variants: MGN, PI-MGN (physics loss), In-MGN (inflow and acceleration features), In-PI-MGN (both)	Node features: velocity u, du/dt, inflow information, node type; ~20,000 nodes per mesh, >75 million training tokens	MGN and PI-MGN (internal baselines); ablation isolating inflow and acceleration features	95 training vs. 10 test geometries; 1-step and 50-step rollout RMSE, globally and by region (bulge, neck, artery); 20 epochs on NVIDIA L4 GPUs	In-PI-MGN: 1-RMSE 0.85 mm/s; 50-RMSE 7.58 mm/s (vs. MGN 1.11 and 50.51→6× better rollout). By region (50-RMSE): bulge 11.87, neck 8.38, artery 4.66. Mass conservation: mean inlet–outlet difference 3.7% (CFD 2.3%). TAWSS error < 10% across the test set	Yes, without fine-tuning: 6 unseen inflow profiles (ICA and MCA) with good performance, and 4 zero-shot out-of-distribution patient-specific geometries (spatial distribution reproduced; magnitude discrepancies in 2 cases)	Near-real-time inference vs. ~1 h per CFD run (~20 h training; break-even after ~20 simulations); physics constraints are decisive for rollout stability; releases the open BenchAnXplore benchmark	Semi-idealized geometries sharing one parent artery; rigid walls and Newtonian blood in the reference CFD; fixed timestep with untested temporal generalization; only 4 patient-specific cases; clinical validation still needed
Liao 2023 [60]	Post-embolization recurrence (recanalisation)	Recanalization = increase in Raymond–Roy grade or within-grade enlargement leading to retreatment; stable = no change on ≥1 y MRA follow-up	Vel-PointNet—PointNet fed with morphological coordinates (x, y, z) plus CFD velocity components (u, v, w); compared with conventional PointNet (morphology only)	3D morphological–hemodynamic point clouds from CFD mesh nodes, with and without the parent artery; 0D scalars for ML: max size, height, neck width, VolAveVelRatio	Logistic regression, KNN, linear and Gaussian SVM, gradient boosting (GBC), MLP; conventional PointNet	65% train/35% test; repeated 6-fold cross-validation (100× ML, 1000× DL); AUC, AUPRC, confusion matrix	Training set—Vel-PointNet: AUC 1.000, AUPRC 1.000, sensitivity and specificity 1.000. Best ML: GBC 0.942/0.944; KNN 0.919/0.864. Test—Vel-PointNet (no parent artery): 17/17 stable and 1/2 recanalized correctly. Morphology-only PointNet: AUC 0.787/AUPRC 0.853 (−21.3% AUC)	None	3D morphological–hemodynamic features outperform spatiotemporally averaged 0D scalars; velocity information is the main driver; including the parent artery worsened performance in this setting	Very small, highly imbalanced sample (8 recanalized; 2 in the test set); perfect training AUC suggests overfitting; steady-state (non-pulsatile) CFD; single center; internal numerical inconsistency; no external validation
Pelissier 2026 [61]	Thrombus formation in IAs—multiphysics prediction of velocity, platelet concentrations and thrombus fraction	High-fidelity finite-element multiphysics simulations (CIMLIB-CFD); 6 cardiac cycles simulated, 4 retained for training (10 ms step)	Transformer-based GNN with adjacency masking in multi-head self-attention, in two configurations: one4all (single model, all fields) and one4each (one model per physical field)	Node coordinates, platelet concentrations at t and t − Δt, velocity at t and t − Δt, inlet velocity at t + Δt; ~65,000 nodes per mesh (2.63 billion training tokens)	MeshGraphNet (15 message-passing layers), BSMS-GNN and Transolver++, all trained with matched steps and tuned hyperparameters	80 train/11 validation/10 test; autoregressive rollout over 5 cardiac cycles; 1-RMSE, ALL-RMSE, ALL-RRMSE, thrombus IoU and relative surface-area error	one4each (S) beats state-of-the-art models by ≥32% in ALL-RMSE while being 45% faster than the next-fastest model (BSMS-GNN) and using 21% fewer parameters; it also predicts thrombus-formation timing correctly, whereas one4all shows a slight delay	Yes, for unseen boundary conditions: three modified inflow profiles and a longer cardiac-cycle count. All models degrade—one4all velocity ALL-RMSE ×5; one4each up to 6× worse for platelet concentrations	Transformer-based GNNs reach state-of-the-art accuracy for IA thrombosis at lower cost; training one model per physical field improves accuracy and enables parallelization; models generalize beyond the training time horizon	2D simulations—extension to 3D is a necessary next step; generic (non-patient-specific) inflow profiles; randomized reactive surface rather than a real device configuration; degradation under unseen inflows
Sheng 2026 [62]	Peak-systolic 3D velocity field and surface wall shear stress as a CFD surrogate	ANSYS (ANSYS Inc., Canonsburg, PA, USA) Fluent CFD solutions at peak systole; incompressible Newtonian blood, rigid walls, mesh-independence tested (0.4–0.6 M elements)	PointFlowNet—geometry-aware point-cloud network (PointNet++/Point-MAE inspired) with a group encoder (1024 kNN patches), distance-to-wall feature and global max pooling; ~3.53 M parameters	Interior flow-domain points only, with normalized coordinates plus Euclidean distance to the nearest wall (d_wall)—a simpler alternative to a signed distance function	PointNet++, Graph U-Net and the dual-input PointNet of Li et al., with and without d_wall; internal ablations	8:1:1 split of 984 geometries, up to 200 epochs, 3 seeds; MAE, NMAE, rL2; Bland–Altman; zero-shot OOD and LOOCV	Velocity (whole geometry): MAE 0.065 m/s, NMAE 4.05%, rL2 19.2%; aneurysm region 4.76%/23.4%. WSS: MAE 4.22 Pa, NMAE 2.59%, rL2 23.9%. Baselines with d_wall: Graph U-Net 4.19%, Li (norm) 4.55%, PointNet++ 5.22%. Removing d_wall is the largest ablation loss (4.05% → 4.70%). Inference ~1.6 s (velocity) and ~0.3 s (WSS) per case	Zero-shot on 5 non-idealized (patient-derived) geometries degrades markedly (NMAE 19.1%, rL2 62.4%); LOOCV fine-tuning improves it to 10.8%/37.0%. No true clinical external validation	A point-cloud network with a distance-to-wall feature achieves high near-real-time accuracy on idealized MCA bifurcation aneurysms, with d_wall the dominant contributor; a clear generalization gap to patient-specific anatomy remains	Synthetic idealized geometries only; peak systole only (time-dependent indices such as OSI not estimable); rigid walls, Newtonian fluid, fixed boundary conditions; no clinical external validation
Shou 2024 [63]	Rupture status (ruptured vs. unruptured)	Clinical rupture status of IAs reconstructed from 3D rotational DSA	PointNet-based model extracting hemodynamic cloud features at 16, 32, 64 and 1024 dimensions, coupled to machine-learning classifiers	Group A: geometric + hemodynamic parameters. Groups B1–B4: A + hemodynamic cloud features of 1024, 64, 32 and 16 dimensions	XGBoost, CatBoost, SVM, LightGBM and logistic regression across groups A and B1–B4	10-fold cross-validation; AUC, accuracy, sensitivity, specificity, ROC curves	Group B4 (16-D)—LR: accuracy 0.908, sensitivity 0.851, specificity 0.929, AUC 0.944. SVM highest AUC 0.946 (accuracy 0.854, sensitivity 0.518, specificity 0.985). XGBoost 0.887/0.917. Group A—LR 0.885/0.921	None	Combining PointNet-derived hemodynamic cloud features with ML improves rupture-status classification; low-dimensional (16-D) features outperformed 1024-D ones, suggesting compression reduces redundancy and overfitting	Small, imbalanced sample (149 IAs, 40 ruptured) with low sensitivity in several models (0.44–0.85); simplifying CFD assumptions; single center; no external validation or comparison with clinical scores
Wen 2023 [64]	Prediction of unsteady, nonlinear flows on unstructured meshes (u, v, w, pressure, concentration)—3D aneurysm blood flow is one test case	Numerical (CFD) simulation results; 300 snapshots of aneurysm flow at Re ≈ 100	SGC-DNN—spectral-domain graph convolutional deep neural network using Laplacian eigenvectors as modes, coupled to a DNN modeling each mode’s temporal evolution	Flow-field snapshots at unstructured mesh nodes, organized as a graph via boundary-constrained KNN; learnable diagonal matrix approximated by a 5th-order polynomial	Dynamic mode decomposition (DMD) and a conventional DNN on the same datasets	Cylinder flow (Re 1000–500,000): first 150 timesteps for training. Aneurysm: 200 training vs. 100 test snapshots. Metrics: relative L2 error and R^2^	Aneurysm (relative L2 error)—SGC-DNN: c 0.070, u 0.065, v 0.084, *p* 0.095, w 0.088. DNN: 0.093/0.085/0.112/0.133/0.119. DMD: 0.284/0.223/0.208/0.314/0.267. Cylinder: relative error < 5% and R^2^ > 99.8% in all cases	Not applicable (numerical-methods study, no clinical cohort)	SGC-DNN preserves node adjacency on unstructured meshes, avoiding interpolation error and loss of spatial structure; it outperforms DMD and a plain DNN on complex, irregular internal flows such as aneurysmal blood flow	Non-clinical study with no patient outcomes; a single aneurysm geometry at a single Reynolds number; largest errors for pressure and at the aneurysm inlet; eigendecomposition cost scales with mesh size
Zhou 2025 [65]	Rupture risk (ruptured vs. unruptured status) and effect on clinicians’ diagnostic performance	Labeled ruptured when SAH was present at presentation and confirmed by DSA, CTA or surgery; manual aneurysm segmentation on every CTA slice	GN-Net—two parallel branches: a geometric branch (geometric DL on aneurysm and parent artery) and a neighborhood-aware branch (3D CNN + Transformer encoders on a 48 × 48 × 48 CTA crop), fused in an MLP with softmax	Geometric meshes/point clouds of aneurysm and parent artery; cropped CTA volume for anatomical context; traditional and radiomic morphological features used in comparators	SVM and LR with traditional (TMF), radiomic (RMF) and combined features; Kim et al. (2D); M3T; DAResUNET (enc, unet); TransIAR; GN-Net branch ablations	Train 100 + 100; balanced test (41 + 41), imbalanced test (208/41) and multicenter external test (57 IAs, 4 hospitals); reader study with two 2-week washout periods; 95% CIs reported	Balanced test—GN-Net: accuracy 91.46% (95% CI 85.41–97.51), precision 92.50%, recall 90.24%, AUC 94.59%, AUPR 94.97%, F1 91.36% (TransIAR 89.02%/92.15%; SVM-TMF 79.27%/85.60%). Imbalanced test: accuracy 82.33%, AUC 92.53%. Ablation: neighborhood branch alone 86.59%, geometric branch alone 81.71%	Yes—57 IAs from 4 additional hospitals, different scanners, entirely unseen patients: accuracy 85.96%, precision 96.77%, recall 81.08%, AUC 94.32%, F1 88.24%, outperforming all comparators	Combining local aneurysm/parent-artery geometry with perianeurysmal anatomical context beats the state of the art across three test sets; in the reader study (2 radiologists, 2 neurosurgeons) model assistance raised diagnostic accuracy by ~7% and mean specificity from 73.17% to 80.49% (*p* = 0.0120), benefiting less-experienced readers most (up to +10.50%)	MCA aneurysms only; internal cohort imbalanced towards ruptured (~5×); rupture status used as a surrogate for prospective risk; single-center training with ~500 valid cases; demographics and comorbidities not reported

Additionally, 3DRA/3D-RA, three-dimensional rotational angiography; AR, aspect ratio; AUC, area under the ROC curve; AUPR/AUPRC, area under the precision-recall curve; CFD, computational fluid dynamics; CI, confidence interval; CNN, convolutional neural network; CTA, computed tomography angiography; DL, deep learning; DSA, digital subtraction angiography; DSC, Dice similarity coefficient; GNN, graph neural network; IA, intracranial aneurysm; ICA, internal carotid artery; IoU, intersection over union; KNN, k-nearest neighbor; LOOCV, leave-one-out cross-validation; LR, logistic regression; MAE, mean absolute error; MCA, middle cerebral artery; ML, machine learning; MLP, multilayer perceptron; MRA, magnetic resonance angiography; NMAE, normalized MAE; NSI, non-sphericity index; OOD, out-of-distribution; OSI, oscillatory shear index; RF, random forest; rL2, relative L2 error; RMSE, root mean square error; SAH, subarachnoid hemorrhage; SD, standard deviation; SHAP, SHapley Additive exPlanations; SVM, support vector machine; TAWSS, time-averaged WSS; WSS, wall shear stress.

**Table 3:** Generalization of graph and geometric deep-learning models for intracranial aneurysms: studies reporting external validation, transfer testing, or out-of-distribution testing.

Study	Task	Generalization Test Performed	Test Set	Performance in Development Cohort	Performance on External/OOD Test	Change	Comparator on the Same Data
**A. Independent external validation in a separate clinical cohort**							
Cao 2024 [55]	Rupture status. PointNet++ on the surface, dome-only and parent-artery-preserving cut1 configurations.	External clinical cohort: an independent recruitment project within the same open database, with no patient overlap.	159 IAs (87 ruptured, 72 unruptured), @neurIST project; 3DRA; same standardized segmentation and re-meshing pipeline as the development set.	AUC 0.85 (cut1) AUC 0.81 (dome) Accuracy 0.82, sensitivity 0.72, specificity 0.86 (cut1)	AUC 0.71 (cut1) AUC 0.69 (dome)	−0.14 (cut1) −0.12 (dome)	PCA-based LASSO of the database authors: AUC 0.82 internally, 0.67 externally. On the external set the geometric model therefore outperformed the morphological one (0.71 vs. 0.67).
Choi 2025 [57]	Rupture status. Automated nnU-Net segmentation with two pretrained PointNet++ stages, feeding a random forest.	External clinical cohort with a change in imaging modality (CTA → 3D-RA) and an independent segmentation pipeline.	Aneurisk public dataset (Niguarda Ca’ Granda, Milan), 3D-RA. A total of 25 cases provided all the information required, although the abstract inconsistently states 24 IAs (10 ruptured, 15 unruptured).	AUC 0.851 Accuracy 0.782, sensitivity 0.804, specificity 0.760	AUC 0.805 Accuracy 0.7917, recall 0.625, specificity 0.875	AUC −0.046 Recall −0.179	PHASES on the same internal data: AUC 0.700. PHASES could not be computed on Aneurisk for want of clinical covariates, so no external comparator exists.
Zhou 2025 [65]	Rupture status. GN-Net, fusing a geometric branch with a 3D convolutional and transformer branch over the perianeurysmal volume.	External clinical cohort: multicenter, entirely unseen patients and scanners.	57 IAs (37 ruptured, 20 unruptured) from four additional hospitals; largely inherited from a published comparator study, to which 14 cases were added.	Accuracy 91.46% AUC 94.59% F1 91.36%	Accuracy 85.96% AUC 94.32% F1 88.24%	Accuracy −5.50 pp AUC −0.27 pp	Best published comparator (TransIAR) on the same external set: accuracy 84.21%, AUC 90.14%. GN-Net was superior on every external metric.
**B. Out-of-distribution and transfer testing of hemodynamic surrogates (no clinical cohort; error metrics, so lower is better)**							
Lannelongue 2026 [59]	Time-resolved 3D velocity field. Physics-constrained MeshGraphNet, In-PI-MGN, on the volumetric simulation mesh.	Two out-of-distribution tests: (i) unseen inflow waveforms; (ii) zero-shot inference on patient-specific anatomy absent from training.	(i) The three test cases re-simulated at alternative inflow rates. (ii) Four patient-specific ICA and MCA geometries from an internal database: inlet 2.5–4.8 mm, sac 4.5–15.0 mm, against 4.0 mm and 3–9 mm in training.	50-step rollout RMSE 7.58 mm/s; single-step 0.85 mm/s Relative velocity error 1.6% TAWSS error < 10%	(i) Inflow, six unseen profiles with no retraining: 50-step RMSE 14.3 mm/s, single-step 2.4 mm/s; fields remained qualitatively close to ground truth. (ii) Anatomy: relative velocity error 11%, with order-of-magnitude discrepancies in 2 of the 4 cases.	Anatomy: × 6.9 (relative error) Inflow: × 1.9 (50-step RMSE)	Unconstrained MeshGraphNet baseline: 50-step rollout RMSE 50.51 mm/s. No non-learned comparator on the zero-shot cases.
Pelissier 2026 [61]	Coupled velocity, platelet concentration and thrombus fraction. Transformer-based GNN with adjacency-masked self-attention.	Two OOD tests, both on the same test geometries: (i) three modified inflow waveforms; (ii) rollout extended from five to nine cardiac cycles. No unseen anatomy was tested.	The 10 held-out geometries, re-simulated under Inflow A (delayed systolic peak), B (double systolic spike) and C (double spike, cycle lengthened 0.8 s → 0.96 s).	ALL-RMSE at least 32% below the best published comparator, with 21% fewer parameters and 45% faster inference (one4each configuration).	All configurations degraded. The one-model-per-field (one4each) configuration degraded moderately; the multitask (one4all) configuration degraded severely on velocity.	Multitask velocity ALL-RMSE × 5	Mesh-based comparators (MeshGraphNet, BSMS-GNN) under the original inflow only; not re-evaluated under the modified waveforms.
Sheng 2026 [62]	Peak-systolic velocity and wall shear stress. Geometry-aware point-cloud network with an explicit distance-to-wall feature.	Zero-shot inference under geometric domain shift, onto non-idealized geometries withheld from training.	Five non-idealized synthetic geometries, generated in an earlier study from patient-specific shapes, registered to the training frame and simulated under the same boundary conditions.	Velocity NMAE 4.05%, relative L2 19.2% (full domain) Within the sac: NMAE 4.76%, rL2 23.4% WSS NMAE 2.59%	Zero-shot velocity NMAE 19.1%, relative L2 62.4%	NMAE × 4.7 rL2 × 3.3	Best ablated variant without the distance-to-wall feature: NMAE 4.70% internally. No comparator evaluated zero-shot.

IA, intracranial aneurysm; AUC, area under the receiver operating characteristic curve; NMAE, normalized mean absolute error; rL2, relative L2 error; RMSE, root mean square error; TAWSS, time-averaged wall shear stress; OOD, out-of-distribution; pp, percentage points; CTA, computed tomography angiography; 3DRA/3D-RA, three-dimensional rotational angiography; ICA, internal carotid artery; MCA, middle cerebral artery; GNN, graph neural network; LASSO, least absolute shrinkage and selection operator; PCA, principal component analysis; PHASES, Population, Hypertension, Age, Size of aneurysm, Earlier subarachnoid hemorrhage, and Site of aneurysm score.

**Table 4:** Heterogeneity of computational fluid dynamics assumptions and hemodynamic outputs across included studies.

Study (Ref.)	Simulation Type	Solver/Numerical Platform	Blood Rheology	Wall Assumption	Inlet Boundary Condition	Outlet Boundary Condition	Mesh/Geometric Domain	Temporal Regime (Cardiac Cycles)	Principal Hemodynamic Output Used by the Learning Model	Main Limitation for Cross-Study Comparability
Chen 2022 [56]	CFD, laminar, transient, rigid wall	ANSYS Fluent 18.0; meshing in ANSYS ICEM 18.0; post-processing in CFD-Post	Newtonian, incompressible; ρ = 1060 kg·m^−3^, μ = 0.004 N·s·m^−2^	Rigid, no-slip	Pulsatile velocity waveform obtained by transcranial Doppler in a single healthy subject and applied to every case	Zero pressure gradient at the outlets	Patient-specific 3DRA/rotational DSA geometries; element size 0.25 mm; boundary-fitted prism layers with node-spacing growth ratio 1.3	Transient; two cardiac cycles of 0.8 s; Δt = 0.01 s; systolic phase of the second cycle exported	Nodal WSS, OSI, pressure and velocity fields (“hemodynamic clouds”) encoded by PointNet into 1024-dimensional features, plus 18 scalar hemodynamic parameters including HOA, LSA and energy loss	Single generic inflow waveform; rigid wall and Newtonian rheology; only one instant of the cycle exported; no mesh-independence or sensitivity analysis reported
Liao 2023 [60]	CFD, steady state, rigid wall	ANSYS CFX 16.2; meshing in ANSYS ICEM CFD 16.2	Newtonian; ρ = 1100 kg·m^−3^, μ = 0.0036 Pa·s	Rigid, no-slip	Mass flow rate of 0.003465 kg·s^−1^, taken from a published pulsatile profile at end diastole; identical for all patients	Static pressure of 0 Pa	Patient-specific 3D-RA geometries of ICPC aneurysms, pre-treatment; inlet and outlet extended to obtain fully developed flow; seven prism layers at the wall; element size and mesh-independence NR	Steady state; no cardiac cycle simulated	Velocity components (u, v, w) at mesh nodes inside the aneurysm sac, fed to Vel-PointNet, plus dimensionless velocity, shear-rate, inflow-rate and turnover-time ratios	Single steady-state solution at one point of the cardiac cycle, so no OSI or cycle-averaged descriptor is defined; boundary conditions uniform across patients (acknowledged by the authors); simulation performed on the untreated geometry although the endpoint is post-embolization recanalization
Shou 2024 [63]	CFD, laminar, transient, rigid wall	ANSYS Workbench Fluent 18.0; post-processing in CFD-Post; surface models prepared in Geomagic	Newtonian, incompressible; ρ = 1055 kg·m^−3^, μ = 0.004 kg·m^−1^·s^−1^	Rigid, no-slip	Inlet velocity prescribed as a function of space and time through a user-defined function, derived from Doppler ultrasonography; per-patient measurement not documented	Static pressure with no pressure gradient at the outlets	Patient-specific 3D rotational DSA geometries; mesh element size, mesh type and mesh-independence study NR	Transient; two cardiac cycles of 0.8 s; time interval 0.01 s; systolic portion of the second cycle exported	Velocity, wall pressure, WSS and OSI clouds encoded by PointNet into 16-, 32-, 64- and 1024-dimensional features	Mesh not described; inflow not demonstrably patient-specific; rigid wall and Newtonian rheology; only the systolic portion of the second cycle analyzed
Wen 2023 [64]	CFD data reused from a public repository (unsteady, three-dimensional); the aneurysm is one of three test cases of a methodological paper	NR for the aneurysm case; the flow data are publicly available from an external repository. The OpenFOAM URANS set-up described in the paper refers to the circular-cylinder benchmark, not to the aneurysm case	NR	NR	NR	NR	Unstructured grid over a single intracranial (ICA) aneurysm region; node and cell counts NR; Re ≈ 100	Unsteady and without evident periodicity; 300 stored snapshots (200 training, 100 testing); number of cardiac cycles NR	Velocity components u, v, w, pressure and a transported scalar concentration on the simulation graph	Solver, rheology, wall and boundary conditions of the aneurysm simulation are not reported; single geometry; no WSS or cycle-averaged descriptor available
Hervé 2026 [58]	CFD/FSI data reused from the public AnXplore corpus; no simulation performed by the authors	NR in the article (simulations attributed to the source dataset)	NR in the article	NR in the article; the source corpus is described as a fluid–structure interaction study, but the variant used is not specified	NR in the article	NR in the article	101 AnXplore models: aneurysm domes segmented from the IntrA/Intra3D dataset and transplanted onto a single uniform parent vessel, so that geometric variation is confined to the sac; mesh characteristics NR	NR; performance reported as single-step and all-rollout RMSE over the stored timeframes	Time-resolved velocity field predicted on the simulation mesh (all-rollout RMSE)	No simulation parameter is reported in the article itself, so the reference standard is only traceable through the source dataset; all geometries share one idealized parent vessel
Lannelongue 2026 [59]	CFD, transient, three-dimensional, rigid wall	In-house finite element C++ library solving a mixed formulation of the incompressible Navier–Stokes equations with a variational multiscale method	Non-Newtonian, shear-thinning; a Carreau–Yasuda type law is presented as the constitutive model (Casson also cited); numerical parameter values NR	Rigid, no-slip; explicitly justified as adequate when the interest is flow pattern and shear-derived biomarkers rather than wall compliance	Fully developed parabolic pulsatile mass-flow profile derived from published ICA measurements, preceded by a 0.2 s linear ramp; scaled to a peak systolic flow of ≈6 mL·s^−1^ (peak Re ≈ 400); identical for all geometries	Resistive boundary condition emulating the downstream vasculature, tuned to a physiological pressure variation of ≈40 mmHg over the cycle	105 semi-idealized geometries: aneurysm sacs derived from IntrA placed on a parent vessel of fixed 4 mm diameter. High-fidelity meshes with boundary-layer refinement (≈200,000 nodes, ≈1 M elements); unstructured tetrahedra with 0.3 mm target size selected after testing 0.15–0.4 mm; a lighter interpolated mesh (<25,000 nodes, 120,000 elements) used for training	Transient; two cardiac cycles of 0.8 s with Δt = 0.001 s, the first cycle discarded as initialization; 80 timeframes per case stored at 0.01 s	Transient velocity and pressure fields on the mesh, with WSS and time-averaged WSS derived from them	Semi-idealized anatomy with a single fixed parent-vessel diameter; generic inflow; rigid wall; training performed on an interpolated coarse mesh rather than the high-fidelity mesh
Sheng 2026 [62]	CFD, transient, rigid wall	ANSYS Fluent 2023 R2 (finite volume); meshing in ANSYS Workbench 2023 R2; export with ANSYS CFD-Post 2023 R2	Newtonian, incompressible; ρ = 1060 kg·m^−3^, μ = 3.5 × 10^−3^ Pa·s	Rigid, no-slip	Periodic pulsatile inflow waveform from the literature, peak inlet mass flow 0.0048 kg·s^−1^; identical for all geometries	Pressure waveforms prescribed at the two outlets; identical for all geometries	984 parametrically generated MCA bifurcation aneurysms (no patient-derived anatomy in training); unstructured tetrahedral mesh with prism boundary layers; mesh-independence test giving 0.4–0.6 M elements; domain truncated to the sac plus 7 mm of each adjoining vessel	Transient; four cardiac cycles of 0.8 s with Δt = 0.001 s; final cycle retained; only the peak systolic instant used	Peak-systolic three-dimensional velocity field at interior points and three-component surface WSS	Entirely synthetic parametric anatomy; single set of boundary conditions shared by all 984 cases; only the peak systolic instant modeled, so no OSI or cycle-averaged descriptor; rigid wall and Newtonian rheology, acknowledged by the authors
Pelissier 2026 [61]	Multiphysics: incompressible Navier–Stokes coupled to convection–diffusion–reaction equations for platelet transport, agonists and thrombin generation; two-dimensional	In-house finite element code CIMLIB-CFD with VMS-stabilized formulation, on an HPC cluster (2048 cores)	Non-Newtonian, shear-thinning; Carreau–Yasuda model (ρ = 1056 kg·m^−3^, μ_0_ = 0.0456 Pa·s, μ∞ = 0.0032 Pa·s, λ = 10.03 s, n = 0.344)	Rigid; no fluid–structure interaction. The growing platelet plug is represented as an immersed rigid body through a level-set formulation	Pulsatile parabolic velocity profile scaled to a mean ICA flow rate of 4 mL·s^−1^, preceded by a 0.2 s linear ramp	Linear hydraulic resistance model P(t) = P_0_ + R_d·Q_out representing the distal vasculature (P_0_ = −3.7 kPa, R_d = 1.31 kPa·s·mL^−1^)	Two-dimensional cross-sections of 101 patient-derived AnXplore geometries; uniform element size 0.05 mm; ≈65,000 nodes per geometry; randomized reactive surface of two consecutive boundary edges of the aneurysm bulge	Transient; six cardiac cycles of 0.8 s (5 s simulated) plus a 0.2 s ramp; Δt = 10^−3^ s with frames stored every 10 steps; first cycle discarded, following four used for training	Velocity field, unactivated and activated platelet concentrations and thrombus fraction over time	Two-dimensional domain; thrombogenic surface randomized rather than patient-specific; rigid wall despite the fluid–structure interaction origin of the source geometries; outputs are not commensurable with the WSS/OSI-based outputs of the other studies

NR, not reported; 3DRA/3D-RA, three-dimensional rotational angiography; CFD, computational fluid dynamics; DSA, digital subtraction angiography; FSI, fluid–structure interaction; HOA, high oscillatory area; HPC, high-performance computing; ICA, internal carotid artery; ICPC, internal carotid-posterior communicating artery; LSA, low shear area; MCA, middle cerebral artery; OSI, oscillatory shear index; Re, Reynolds number; RMSE, root mean square error; URANS, unsteady Reynolds-averaged Navier–Stokes; VMS, variational multiscale; WSS, wall shear stress.

## Data Availability

All data supporting the reported results are contained within the article and the Supplementary Materials. The full search strategies, the data extraction template and the risk of bias judgements are available from the corresponding author on reasonable request. No new data was created.

## Supplementary Materials

The following supporting information can be downloaded at: https://www.mdpi.com/article/10.3390/life16091538/s1, Table S1. PICO(TS) framework and eligibility criteria applied in the review; Table S2. Full electronic search strategies, reported in accordance with PRISMA-S. All searches were run on 29 July 2026 with no language, date or publication type limit at the search stage; Table S3: Risk of bias and applicability assessment using PROBAST, supplemented by the signalling items developed for prediction models based on artificial intelligence, with reference standard heterogeneity added as a prespecified fifth domain; Table S4: Reporting completeness of the included studies against selected TRIPOD+AI items, together with transparency items relevant to reproducibility. Table S5: PRISMA 2020 checklist.

## Abbreviations

AUC, area under the receiver operating characteristic curve; AUPRC, area under the precision recall curve; CFD, computational fluid dynamics; CTA, computed tomography angiography; DSA, digital subtraction angiography; ELAPSS, Earlier subarachnoid hemorrhage, Location, Age, Population, Size and Shape score; GNN, graph neural network; IA, intracranial aneurysm; MAE, mean absolute error; MRA, magnetic resonance angiography; NMAE, normalized mean absolute error; OSI, oscillatory shear index; PHASES, Population, Hypertension, Age, Size, Earlier subarachnoid hemorrhage and Site score; PRISMA, Preferred Reporting Items for Systematic Reviews and Meta-Analyses; PROBAST, Prediction model Risk Of Bias Assessment Tool; rL2, relative L2 error; RMSE, root mean square error; SWiM, Synthesis Without Meta-analysis; TAWSS, time averaged wall shear stress; TRIPOD, Transparent Reporting of a multivariable prediction model for Individual Prognosis Or Diagnosis; UIATS, Unruptured Intracranial Aneurysm Treatment Score; WSS, wall shear stress; 3DRA, three dimensional rotational angiography.
