# Supplementary material for: Graph and Geometric Deep Learning for Intracranial Aneurysm Geometry and Hemodynamics: A Systematic Review with Implications for Neurovascular Implant Design and Evaluation
Source: Life (Basel). 2026 Sep 15;16(9):1538. doi: 10.3390/life16091538 (PMC13608527; doi:10.3390/life16091538)
Supplement: Supplementary file 1 [file life-16-01538-s001.zip › life-4503817-supplementary.pdf]

Supplementary Materials

Graph and Geometric Deep Learning for Intracranial Aneurysm Geometry and Hemodynamics: A Systematic Review with Implications for Neurovascular Implant Design and Evaluation

Rudolfh Batista Arend, Bruno Zilli Peroni, Natan Lucca Lima, Miguel Cruz Garcia, Rafael Torres Fonseca dos Santos, Daniel Kerpel, Gustavo Simiano Jung, Alex Roman, Guilherme Gago, Martin Batista Coutinho da Silva, Antonio Delacy Martini Vial and Edoardo Agosti

This file contains Tables S1 to S5 supporting the systematic review. Tables S1 and S2 document the eligibility framework and the full electronic search strategies. Tables S3 and S4 present the risk of bias and reporting completeness appraisals. Table S5 is the PRISMA 2020 checklist.

Table S1. PICO(TS) framework and eligibility criteria applied in the review.

Element	Definition	Excluded
**P, Population**	Human intracranial (cerebral) aneurysms, ruptured or unruptured, represented as patient-specific 3D vascular models derived from CTA, MRA, 3D rotational angiography or DSA. In-silico cohorts of patient-derived geometries are eligible. Idealised or synthetic geometries are eligible only when reported alongside patient-derived cases, and are recorded separately.	Extracranial aneurysms (abdominal/thoracic aortic, peripheral) and non-aneurysmal vascular disease, reserved for a pre-specified sensitivity analysis.
**I, Intervention**	Any graph-based deep-learning model applied to the aneurysm geometry or its computational mesh: message-passing GNN, graph convolutional network, graph attention network, graph transformer, MeshGraphNet-type simulators, gauge- or SE(3)-equivariant mesh networks, geometric deep learning on point clouds, and physics-constrained variants. The model must output a haemodynamic field or parameter, or a rupture-related risk estimate.	CNN, U-Net, multilayer perceptron and classical machine-learning models with no graph component (these are eligible only as comparators).
**C, Comparator**	(a) High-fidelity CFD (finite-volume/finite-element) simulation of the same geometry, as reference standard for haemodynamic accuracy; and/or (b) established clinical or morphological risk instruments (PHASES, ELAPSS, UIATS, size/location); and/or (c) non-graph machine-learning baselines; and/or (d) observed rupture status.	Studies with no quantitative comparison against any reference.
**O, Outcomes**	Primary: agreement with the CFD reference for haemodynamic fields and derived parameters (WSS, TAWSS, OSI, RRT, velocity, pressure), expressed as R^2, NMAE, RMSE, correlation or spatial-overlap metrics; and, where reported, discrimination for rupture status or rupture/growth events (AUC, sensitivity, specificity, calibration). Secondary: inference time versus reference CFD; generalisation to unseen geometries and inflow conditions; training-set size; external validation; code and data availability.	Qualitative or purely visual comparison with no reported performance metric.
**T, Timing**	Any; practically bounded from 2017 (introduction of modern message-passing GNN architectures) to the search date. No language restriction.	—
**S, Study design**	Any original prediction-model development and/or validation study: retrospective patient cohorts, in-silico benchmark studies, and methodological papers reporting quantitative performance.	Narrative and systematic reviews, editorials, commentaries, and conference abstracts without extractable performance data.

CTA, computed tomography angiography; DSA, digital subtraction angiography; MRA, magnetic resonance angiography; OSI, oscillatory shear index; RRT, relative residence time; TAWSS, time averaged wall shear stress; WSS, wall shear stress; 3DRA, three dimensional rotational angiography.

Table S2. Full electronic search strategies, reported in accordance with PRISMA-S. All searches were run on 29 July 2026 with no language, date or publication type limit at the search stage.

Database	Interface, date and fields	Search string as executed	Records
**PubMed and MEDLINE**	PubMed web interface<br>29 July 2026<br>Field tags: [Mesh], [tiab]	("Intracranial Aneurysm"[Mesh] OR "intracranial aneurysm*"[tiab] OR "cerebral aneurysm*"[tiab] OR "brain aneurysm*"[tiab] OR "cerebral artery aneurysm*"[tiab] OR "saccular aneurysm*"[tiab] OR "berry aneurysm*"[tiab] OR "aneurysmal subarachnoid hemorrhage"[tiab] OR "aneurysmal subarachnoid haemorrhage"[tiab])<br>AND<br>("graph neural network*"[tiab] OR "graph convolutional network*"[tiab] OR "graph convolution*"[tiab] OR "graph attention network*"[tiab] OR "graph transformer*"[tiab] OR "message passing"[tiab] OR "MeshGraphNet*"[tiab] OR "mesh neural network*"[tiab] OR "mesh convolution*"[tiab] OR "geometric deep learning"[tiab] OR "equivariant neural network*"[tiab] OR "gauge equivariant"[tiab] OR "point cloud*"[tiab] OR "PointNet*"[tiab] OR "vessel graph*"[tiab] OR GNN[tiab] OR GCN[tiab] OR GAT[tiab])<br>AND<br>("Hemodynamics"[Mesh] OR "Hydrodynamics"[Mesh] OR "Rupture, Spontaneous"[Mesh] OR "computational fluid dynamic*"[tiab] OR CFD[tiab] OR hemodynamic*[tiab] OR haemodynamic*[tiab] OR "wall shear stress"[tiab] OR WSS[tiab] OR TAWSS[tiab] OR "oscillatory shear index"[tiab] OR OSI[tiab] OR "relative residence time"[tiab] OR "Navier-Stokes"[tiab] OR "flow field*"[tiab] OR "surrogate model*"[tiab] OR "reduced order model*"[tiab] OR ruptur*[tiab] OR "risk stratification"[tiab] OR PHASES[tiab] OR ELAPSS[tiab] OR UIATS[tiab])	20
**Embase**	Embase.com<br>29 July 2026<br>Field tags: /exp, :ti,ab,kw	('brain aneurysm'/exp OR 'intracranial aneurysm*':ti,ab,kw OR 'cerebral aneurysm*':ti,ab,kw OR 'brain aneurysm*':ti,ab,kw OR 'saccular aneurysm*':ti,ab,kw OR 'berry aneurysm*':ti,ab,kw OR 'aneurysmal subarachnoid hemorrhage':ti,ab,kw OR 'aneurysmal subarachnoid haemorrhage':ti,ab,kw)<br>AND<br>('graph neural network'/exp OR 'graph neural network*':ti,ab,kw OR 'graph convolutional network*':ti,ab,kw OR 'graph convolution*':ti,ab,kw OR 'graph attention network*':ti,ab,kw OR 'graph transformer*':ti,ab,kw OR 'message passing':ti,ab,kw OR 'meshgraphnet*':ti,ab,kw OR 'mesh neural network*':ti,ab,kw OR 'geometric deep learning':ti,ab,kw OR 'equivariant neural network*':ti,ab,kw OR 'point cloud*':ti,ab,kw OR 'vessel graph*':ti,ab,kw OR gnn:ti,ab,kw OR gcn:ti,ab,kw)<br>AND<br>('computational fluid dynamics'/exp OR 'hemodynamics'/exp OR 'wall shear stress'/exp OR 'aneurysm rupture'/exp OR 'computational fluid dynamic*':ti,ab,kw OR cfd:ti,ab,kw OR hemodynamic*:ti,ab,kw OR haemodynamic*:ti,ab,kw OR 'wall shear stress':ti,ab,kw OR wss:ti,ab,kw OR tawss:ti,ab,kw OR 'oscillatory shear index':ti,ab,kw OR osi:ti,ab,kw OR 'relative residence time':ti,ab,kw OR 'navier stokes':ti,ab,kw OR 'flow field*':ti,ab,kw OR 'surrogate model*':ti,ab,kw OR ruptur*:ti,ab,kw OR 'risk stratification':ti,ab,kw OR phases:ti,ab,kw OR elapss:ti,ab,kw OR uiats:ti,ab,kw)	14
**Scopus**	Scopus Advanced Search<br>29 July 2026<br>TITLE-ABS-KEY applied to each block	("intracranial aneurysm*" OR "cerebral aneurysm*" OR "brain aneurysm*" OR "cerebral artery aneurysm*" OR "saccular aneurysm*" OR "berry aneurysm*" OR	345

		"aneurysmal subarachnoid hemorrhage" OR "aneurysmal subarachnoid haemorrhage")<br>AND<br>("graph neural network*" OR "graph convolutional network*" OR "graph convolution*" OR "graph attention*" OR "graph transformer*" OR "message passing" OR "meshgraphnet*" OR "mesh neural network*" OR "mesh convolution*" OR "geometric deep learning" OR "equivariant neural network*" OR "gauge equivariant" OR "point cloud*" OR "pointnet*" OR "vessel graph*" OR "GNN" OR "GCN")<br>AND<br>("computational fluid dynamic*" OR "CFD" OR hemodynamic* OR haemodynamic* OR "wall shear stress" OR "WSS" OR "TAWSS" OR "oscillatory shear index" OR "OSI" OR "relative residence time" OR "Navier-Stokes" OR "flow field*" OR "surrogate model*" OR "reduced order model*" OR ruptur* OR "risk stratification" OR "PHASES" OR "ELAPSS" OR "UIATS")	
**Web of Science**	Core Collection, Advanced Search<br>29 July 2026<br>Topic (TS) field	("intracranial aneurysm*" OR "cerebral aneurysm*" OR "brain aneurysm*" OR "cerebral artery aneurysm*" OR "saccular aneurysm*" OR "berry aneurysm*" OR "aneurysmal subarachnoid h$emorrhage")<br>AND<br>("graph neural network*" OR "graph convolutional network*" OR "graph convolution*" OR "graph attention*" OR "graph transformer*" OR "message passing" OR "meshgraphnet*" OR "mesh neural network*" OR "mesh convolution*" OR "geometric deep learning" OR "equivariant neural network*" OR "gauge equivariant" OR "point cloud*" OR "pointnet*" OR "vessel graph*" OR "GNN" OR "GCN")<br>AND<br>("computational fluid dynamic*" OR "CFD" OR hemodynamic* OR haemodynamic* OR "wall shear stress" OR "WSS" OR "TAWSS" OR "oscillatory shear index" OR "OSI" OR "relative residence time" OR "Navier-Stokes" OR "flow field*" OR "surrogate model*" OR "reduced order model*" OR ruptur* OR "risk stratification" OR "PHASES" OR "ELAPSS")	35

Total records retrieved from databases, 414. Duplicates removed before screening, 68. Records screened at title and abstract, 346. Supplementary searching comprised backward and forward citation tracking of all included studies, hand searching of preprint servers and of the proceedings of the principal medical imaging and machine learning conferences, and inspection of the repositories associated with the public aneurysm datasets. All four strategies were peer reviewed against the PRESS 2015 checklist before execution.

Table S3. Risk of bias and applicability assessment using PROBAST, supplemented by the signalling items developed for prediction models based on artificial intelligence, with reference standard heterogeneity added as a prespecified fifth domain.

Study	Participants	Predictors	Outcome	Analysis	Reference standard	Overall risk of bias	Overall applicability
**Bizjak 2021**	High	Low	High	High	Not applicable	High	Low concern
**Cao 2024**	High	Low	Unclear	High	Not applicable	High	High concern
**Chen 2022**	High	High	Unclear	High	High	High	High concern
**Choi 2025**	Unclear	Low	Unclear	High	Not applicable	High	High concern
**Herve 2026**	High	Low	Unclear	Unclear	Unclear	Unclear	High concern
**Lannelongue 2026**	High	Low	Not applicable	Unclear	High	Unclear	High concern
**Liao 2023**	High	High	Low	High	High	High	Unclear
**Pelissier 2026**	High	Low	Not applicable	Low	High	Unclear	High concern
**Sheng 2026**	High	Low	Not applicable	Low	High	Unclear	High concern
**Shou 2024**	High	High	Unclear	High	High	High	High concern
**Wen 2023**	High	Low	Not applicable	Unclear	High	Unclear	High concern
**Zhou 2025**	High	Unclear	Low	Unclear	Not applicable	Unclear	Unclear

Judgements were reached by two reviewers independently and reconciled by discussion. Ratings are low, unclear or high risk of bias, and low, unclear or high concern regarding applicability. The reference standard domain applies only to studies in which a computational fluid dynamics or multiphysics simulation served as the ground truth, and it was rated high whenever the simplifying assumptions of the reference simulation, for example rigid walls, Newtonian rheology, generic inflow waveforms, steady state flow or two dimensional geometry, were adopted without an accompanying sensitivity analysis. The analysis domain was rated high whenever the number of outcome events was small relative to the number of candidate predictors, whenever calibration was not assessed, or whenever no validation independent of the development data was performed. Applicability was rated with respect to the review question, so studies conducted entirely on synthetic or semi-idealised geometries, or without a clinical endpoint, attract high concern even when methodologically sound.

Table S4. Reporting completeness of the included studies against selected TRIPOD+AI items, together with transparency items relevant to reproducibility.

Study	Sample size justified	Missing data reported	Discrimination reported	Calibration reported	Measures of uncertainty	Independent external validation	Code available	Data available
**Bizjak 2021**	No	Not applicable	Yes	No	No	No	No	No
**Cao 2024**	No	Not applicable	Yes	No	No	Yes	No	Yes, public database
**Chen 2022**	No	Not applicable	Yes	No	Yes, 95% CI for AUC	No	No	No
**Choi 2025**	No	Not reported	Yes	No	No	Yes	No	Partly, external set public
**Herve 2026**	No	Not applicable	Yes	Not applicable	No	No	No	Yes, public datasets
**Lannelongue 2026**	No	Not applicable	Not applicable	Not applicable	No	Partly, unseen inflows and zero shot geometries	Not stated	Yes, benchmark released
**Liao 2023**	No	Not applicable	Yes	No	No	No	No	No
**Pelissier 2026**	No	Not applicable	Not applicable	Not applicable	No	Partly, unseen boundary conditions	Not stated	Partly, derived from public geometries
**Sheng 2026**	No	Not applicable	Not applicable	Not applicable	No	Partly, out of distribution geometries	Not stated	No
**Shou 2024**	No	Not applicable	Yes	No	No	No	No	No
**Wen 2023**	No	Not applicable	Not applicable	Not applicable	No	No	No	No
**Zhou 2025**	No	Not reported	Yes	No	Yes, 95% CI	Yes, four hospitals	Yes, public repository	No

Discrimination and calibration are recorded as not applicable for studies whose output is a continuous physical field rather than a probability. Partly indicates that generalisation was tested against conditions withheld from training, for example unseen inflow waveforms or geometries outside the training distribution, but not in an independent clinical cohort. AUC, area under the receiver operating characteristic curve; CI, confidence interval.

Table S5. PRISMA 2020 checklist.

Item	Section and topic	Checklist item	Location where item is reported
**1**	Title	Identify the report as a systematic review.	Title
**2**	Abstract	See the PRISMA 2020 for Abstracts checklist.	Abstract
**3**	Rationale	Describe the rationale for the review in the context of existing knowledge.	Section 1
**4**	Objectives	Provide an explicit statement of the objectives or questions the review addresses.	Section 1, final paragraph
**5**	Eligibility criteria	Specify the inclusion and exclusion criteria and how studies were grouped for the syntheses.	Section 2.2; Table S1
**6**	Information sources	Specify all databases, registers, websites and other sources searched, and the date each was last searched.	Section 2.3; Table S2
**7**	Search strategy	Present the full search strategies for all databases, registers and websites, including any filters and limits used.	Table S2
**8**	Selection process	Specify the methods used to decide whether a study met the inclusion criteria.	Section 2.4
**9**	Data collection process	Specify the methods used to collect data from reports.	Section 2.5
**10a**	Data items, outcomes	List and define all outcomes for which data were sought.	Section 2.2, Outcomes
**10b**	Data items, other variables	List and define all other variables for which data were sought.	Section 2.5
**11**	Study risk of bias assessment	Specify the methods used to assess risk of bias in the included studies.	Section 2.6; Table S3
**12**	Effect measures	Specify for each outcome the effect measures used in the synthesis or presentation of results.	Section 2.8
**13a**	Synthesis methods, eligibility	Describe the processes used to decide which studies were eligible for each synthesis.	Section 2.8
**13b**	Synthesis methods, preparation	Describe any methods required to prepare the data for presentation or synthesis.	Section 2.5, Section 2.8
**13c**	Synthesis methods, tabulation	Describe any methods used to tabulate or visually display results.	Section 2.8; Tables 1 and 2
**13d**	Synthesis methods, results	Describe any methods used to synthesise results and provide a rationale for the choices made.	Section 2.8, narrative synthesis following SWiM; meta-analysis not appropriate
**13e**	Synthesis methods, heterogeneity	Describe any methods used to explore possible causes of heterogeneity among study results.	Sections 2.6 and 2.8, reference standard treated as a distinct domain
**13f**	Synthesis methods, sensitivity	Describe any sensitivity analyses conducted.	Section 2.2, prespecified subgroup of synthetic versus patient derived geometries
**14**	Reporting bias assessment	Describe any methods used to assess risk of bias due to missing results in a synthesis.	Section 4.7
**15**	Certainty assessment	Describe any methods used to assess certainty in the body of evidence for an outcome.	Sections 2.6 and 3.9
**16a**	Study selection, flow	Describe the results of the search and selection process.	Section 3.1; Figure 1
**16b**	Study selection, exclusions	Cite studies that met many but not all inclusion criteria and explain why they were excluded.	Section 3.1; Section 4.6, final paragraph
**17**	Study characteristics	Cite each included study and present its characteristics.	Section 3.2; Table 1
**18**	Risk of bias in studies	Present assessments of risk of bias for each included study.	Section 3.9; Table S3
**19**	Results of individual studies	Present for all outcomes the summary statistics for each study.	Sections 3.4 to 3.7; Table 2
**20a**	Results of syntheses, characteristics	Summarise the characteristics and risk of bias among contributing studies.	Sections 3.2 and 3.9
**20b**	Results of syntheses, summary	Present results of all statistical syntheses conducted.	Sections 3.4 to 3.8, narrative synthesis; no statistical pooling performed
**20c**	Results of syntheses, heterogeneity	Present results of all investigations of possible causes of heterogeneity.	Sections 3.6 and 3.9
**20d**	Results of syntheses, robustness	Present results of all sensitivity analyses conducted.	Sections 3.6 and 3.8
**21**	Reporting biases	Present assessments of risk of bias due to missing results for each synthesis assessed.	Section 4.7
**22**	Certainty of evidence	Present assessments of certainty in the body of evidence for each outcome assessed.	Sections 3.9 and 4.7

**23a**	Discussion, interpretation	Provide a general interpretation of the results in the context of other evidence.	Sections 4.1 to 4.6
**23b**	Discussion, limitations of evidence	Discuss any limitations of the evidence included in the review.	Sections 3.9, 4.4 and 4.5
**23c**	Discussion, limitations of review	Discuss any limitations of the review processes used.	Section 4.7
**23d**	Discussion, implications	Discuss implications of the results for practice, policy and future research.	Sections 4.3, 4.6 and 5
**24a**	Registration and protocol	Provide registration information for the review, including the register name and registration number.	Back matter, Registration
**24b**	Protocol	Indicate where the review protocol can be accessed, or state that a protocol was not prepared.	Section 2.1; back matter
**24c**	Amendments	Describe and explain any amendments to information provided at registration or in the protocol.	Section 2.1; Section 2.7, translational stage classification applied post hoc
**25**	Support	Describe sources of financial or non-financial support and the role of the funders.	Back matter, Funding
**26**	Competing interests	Declare any competing interests of review authors.	Back matter, Conflicts of Interest
**27**	Availability of data, code and other materials	Report which of the following are publicly available and where: template data collection forms, extracted data, analytic code, other materials.	Back matter, Data Availability Statement

Checklist items are reproduced from the PRISMA 2020 statement. Section numbers refer to the numbered sections of the main manuscript. SWiM, Synthesis Without Meta-analysis.
